# HIV-1 pathogenicity and virion production are dependent on the metabolic phenotype of activated CD4+ T cells

**DOI:** 10.1186/s12977-014-0098-4

**Published:** 2014-11-25

**Authors:** Andrea Hegedus, Maia Kavanagh Williamson, Hendrik Huthoff

**Affiliations:** Department of Infectious Diseases, King’s College London, 2nd Floor Borough Wing, Guy’s Hospital, Great Maze Pond, London, SE1 9RT UK

**Keywords:** Human immunodeficiency virus, Metabolism, Glycolysis, Virion assembly, Apoptosis, Glucose, T lymphocytes

## Abstract

**Background:**

HIV-1, like all viruses, is entirely dependent on the host cell for providing the metabolic resources for completion of the viral replication cycle and the production of virions. It is well established that HIV-1 replicates efficiently in activated CD4+ T cells, whereas resting CD4+ T cells are refractory to infection with HIV-1. A hallmark of T cell activation is the upregulation of glycolysis to meet the biosynthetic and bioenergetic needs of cell proliferation and the execution of effector functions by the secretion of cytokines. To date, it has remained unknown if HIV-1 requires the high glycolytic activity of activated T cells to support its replication.

**Results:**

We report that in primary CD4+ T cells, the flux through the glycolytic pathway is increased upon infection with HIV-1. This increase in glycolytic activity does not occur in T cell lines when infected with HIV-1. By providing cells with galactose instead of glucose, the former being a poor substrate for glycolysis, we monitored the effect of preventing glycolysis in CD4+ T cells on virus replication cycle and cell fate. We observed that HIV-1 infected primary CD4+ T cells cultured in galactose have a survival advantage over those cultured in glucose and this coincides with reduced caspase 3 activation and apoptosis in cultures with galactose. T cell lines do not recapitulate this difference in cell death. Finally, we demonstrate that virion production is dependent on glycolysis as cultures containing galactose yield reduced amounts of HIV-1 virions compared with cultures containing glucose.

**Conclusions:**

The replication of HIV-1 in primary CD4+ T cells causes an increase in glycolytic flux of the cell. Glycolysis is particularly required for virion production and additionally increases the sensitivity of the infected cell to virus-induced cell death.

**Electronic supplementary material:**

The online version of this article (doi:10.1186/s12977-014-0098-4) contains supplementary material, which is available to authorized users.

## Background

As obligate intracellular parasites, viruses are dependent on the host for provision of the biosynthetic and bioenergetic resources required for the completion of the viral life cycle. The study of viral exploitation and manipulation of cellular metabolism is an emerging field that has yielded novel insights into viral replication strategies and their therapeutic intervention in recent years [1,2]. Numerous viruses have now been shown to cause significant alterations in the metabolism of the host cell, including hepatitis C, influenza, herpes simplex and human cytomegalovirus [3-7].

The best-studied case of changes to the cellular metabolism in response to viral infection is that of human cytomegalovirus (HCMV). Cells that are infected with HCMV show a greater uptake of glucose [8], elevated glycolytic activity [4], efflux of citrate from the tricarboxylic acid cycle (TCA cycle) [9], enhanced glutaminolysis [10] and increased fatty acid and pyrimidine synthesis [4,6,9]. Together, these studies have elucidated that the production of the enveloped virus HCMV requires additional fatty acid and nucleotide synthesis for the supply of membrane and viral genomes to progeny virions, respectively [2]. These biosynthetic processes are supported by the upregulation of glycolysis and the TCA cycle. Importantly, the increased demand for fatty acid synthesis in infected cells is met by cataplerosis of citrate from the TCA cycle, which is replenished by anaplerosis of glutamine. It has been noted that these changes are similar to the metabolic reprogramming observed upon oncogenesis [2], suggesting that the biosynthetic needs of virus replication are similar to those of proliferating cells.

The human immunodeficiency virus type 1 (HIV-1) replicates most efficiently in activated CD4+ T cells and it is the depletion of this compartment that heralds the onset of AIDS. In contrast, quiescent or resting T cells are refractory to infection by HIV-1 and this is in part due to a block to reverse transcription as a result of the low levels of nucleotides that are present in resting T cells [11-13]. The activation of T cells upon antigen presentation is associated with a dramatic change in the metabolic activity of the cell that is similar to oncogenic transformation. T cell activation is initiated by a burst of increased oxidative phosphorylation, which is followed by a substantial upregulation of glycolysis as well as glutaminolysis [14,15]. Because the upregulation of glycolysis exceeds oxidative phosphorylation in activated T cells, secretion of lactic acid is increased. The upregulation of glycolysis was long thought to support the proliferation and execution of effector functions of the activated T cells. However, recent work by the Pearce group has shown that activation and proliferation of T cells can be supported by oxidative phosphorylation alone, but that the synthesis of cytokines requires glycolytic metabolism [16].

There is mounting evidence that HIV-1 replication is sensitive to, and modulates the activity of, host metabolism. For instance, the recent discovery of the restriction factor SAMHD1 has led to the insight that it hydrolyses deoxynucleotide triphosphates (dNTPs) to levels that are insufficient to support reverse transcription in monocytes, macrophages, dendritic and quiescent CD4+ T cells [17-21]. In addition, a recent metabolic profiling analysis has revealed increased levels of glycolytic intermediates in HIV-1 infected activated CD4+ T cells as well as an increase in glucose uptake [22]. However, dependency of HIV-1 replication on the metabolic activity of activated CD4+ T cells has not previously been demonstrated. We report here that infection with HIV-1 further increases the glycolytic activity of activated primary CD4+ T cells and that preventing glycolysis in infected cells is associated with increased cell survival. Finally, we show that virion production is dependent on the availability of glucose to T cells.

## Results

### Infection with HIV-1 increases the rate of glycolysis of primary CD4+ T cells but not T cell lines

**Figure 1:**
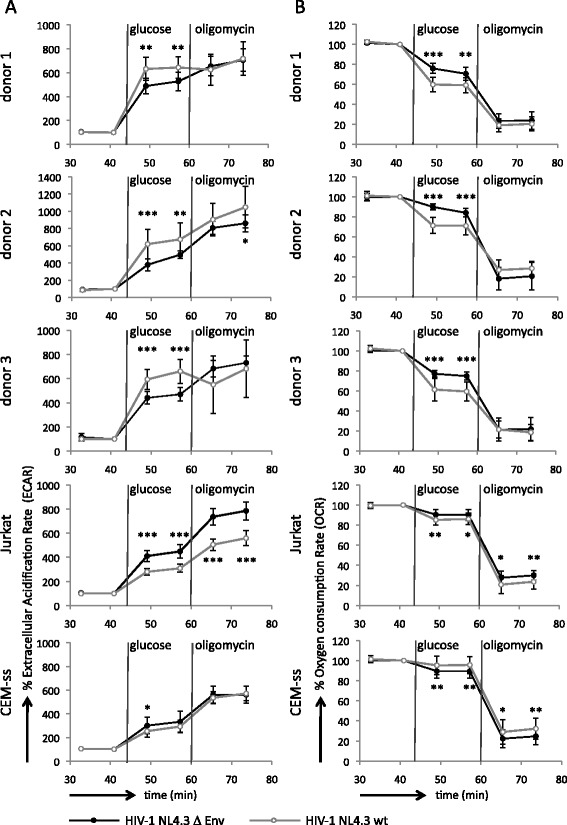
**Monitoring glycolysis and oxidative phosphorylation in HIV-1 NL4.3 infected and uninfected CD4+ T cells upon provision of glucose. A**. The extracellular acidification rate (ECAR) and **B**. oxygen consumption rate (OCR) over time is given as a percentage of the baseline recorded at 41 minutes in the absence of glucose for primary CD4+ T cells from three donors, CEM-ss and Jurkat cells. Vertical lines indicate the injection of glucose and oligomycin, respectively. Data from primary CD4+ T cells represent single plates with 10 wells dedicated to each condition and data for cell lines CEM-ss and Jurkat are the average of two plates. In all cases, infected cell populations were at least 70% positive for HIV-1 p24^Gag^ as determined by flow cytometry. Error bars represent the standard deviation and asterisks indicate p-values from an unpaired t-test: *0.01 < p < 0.05, **0.005 < p < 0.01, ***p < 0.005, data points without an asterisk have non-significant differences.

We first sought to determine if the infection of CD4+ T cells with HIV-1 causes changes in flux through the glycolytic pathway and the TCA cycle. We therefore monitored the extracellular acidification rate (ECAR) as a measure for glycolytic activity and the oxygen consumption rate (OCR) as a measure for oxidative phosphorylation in cultures containing HIV-1 NL4.3 infected cells or cells treated with the non-infectious Env-deleted virus, Δ Env (Figure 1 and Additional file 1). In particular, baseline ECAR and OCR were recorded in glucose-free DMEM with subsequent sequential injections of glucose and the ATP synthase inhibitor oligomycin. As expected, the addition of glucose caused an increase in ECAR in all conditions tested as it is metabolised through glycolysis to produce lactic acid (Figure 1A). This coincides with a general drop in OCR as the utilisation of glucose for ATP production reduces the need for oxidative phosphorylation, known as the Crabtree effect [23] (Figure 1B). The addition of oligomycin causes a further increase in ECAR as upregulation of glycolysis to its maximal capacity compensates for the loss of mitochondrial ATP production, which is evidenced by a drop in OCR to approximately 20% of the baseline.

We observed that the addition of glucose to primary CD4+ T cells infected with HIV-1 NL4.3 consistently resulted in a higher stimulation of ECAR over the baseline than their uninfected counterparts (Figure 1A). While uninfected cells showed an average fourfold induction of the ECAR after addition of glucose, in HIV-1 NL4.3 infected cells the ECAR always exceeded this with an average of a sixfold induction. Indeed, the ECAR of HIV-1 NL4.3 infected cells in the presence of glucose was therefore of a similar magnitude as the maximal ECAR of uninfected cells in the presence of oligomycin. HIV-1 infected primary CD4+ T cells showed little if any increase of the ECAR after the addition of oligomycin (Figure 1A), indicating that the infected cells are operating at or near their maximum glycolytic capacity. In addition, the suppression of the OCR after addition of glucose was more substantial in infected than in uninfected cells, with the OCR dropping to an average of 60% of the baseline instead of 80%, respectively (Figure 1B). Similar results were obtained when cells were infected with the HIV-1 isolate YU2 (Additional file 2). Together, these results demonstrate that primary CD4+ T cells have an increased rate of glycolysis upon infection with HIV-1. This is consistent with a previous study that reported elevated levels of glycolytic intermediates in the cytoplasm of HIV-1 infected cells as well as a moderate increase in glucose uptake compared with uninfected cells [22]. In order to investigate if this may be related to changes in active glucose transport, we assessed the levels of GLUT1 expression in HIV-1 infected and uninfected cells by flow cytometry (Additional file 3). GLUT1 is the main glucose transporter in activated T cells; its expression on the cell surface is upregulated upon T cell activation [24-26] (Additional file 3D) and its silencing by RNAi inhibits HIV-1 replication [27]. In these as well as subsequent experiments, we distinguished three populations of cells: uninfected cells that received an inoculum of Δ Env virions or control supernatant; bystander cells that are the p24^Gag^/GFP/DSredX-negative population of cultures that received wild type or reporter NL4.3 virus; and p24^Gag^/GFP/DSredX-positive cells that are productively infected with HIV-1 NL4.3. We observed no difference in the intracellular expression levels of GLUT1 in HIV-1 NL4.3 infected and uninfected primary CD4+ T cells. When we assessed the surface-expressed levels of GLUT1 in uninfected cells and cells infected with HIV-1 NL4.3-DSredX we noted no large difference between uninfected, bystander and infected cells. However, a small shift in the median fluorescence intensity of the GLUT1-positive cells in the NL4.3-DSredX infected population compared with uninfected and bystander cells was consistently observed. This suggests that there may be a slight increase in the levels of surface-expressed GLUT1 in HIV-1 infected cells (Additional file 3C).

We performed the same extracellular flux experiments with Jurkat and CEM-ss cell lines (Figure 1A and B, and Additional file 2). Unexpectedly, these experiments revealed that neither cell lines are subject to an increased glycolytic rate upon infection with HIV-1. In fact, Jurkat cells infected with HIV-1 NL4.3 showed a lower ECAR than their uninfected counterparts at all conditions tested (Figure 1A). Both Jurkat and CEM-ss cell lines are derived from leukemic T cells [28,29] and it has long been appreciated that cancer causes considerable metabolic changes to cells [30]. Evidently, this extends to the metabolic response to the infection of these cells by HIV-1.

### Manipulating the metabolism of activated T cells by the provision of glucose or galactose

**Figure 2:**
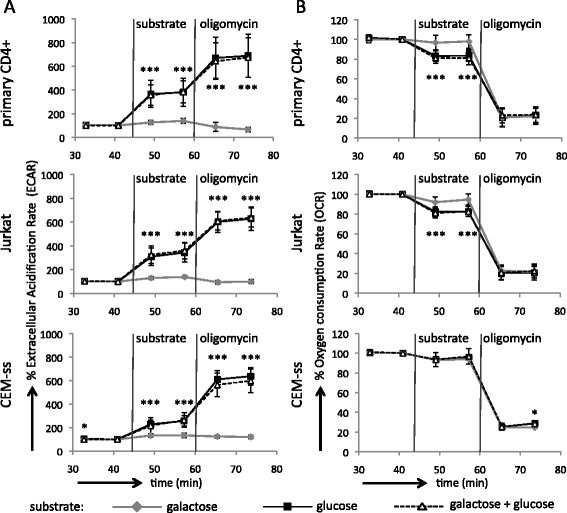
**Galactose is an inefficient substrate for glycolysis in primary CD4+ T cells and T cell lines. A**. The extracellular acidification rate (ECAR) and **B**. oxygen consumption rate (OCR) over time is given as a percentage of the baseline recorded at 41 minutes in the absence of carbohydrate substrate for primary CD4+ T cells from four donors, CEM-ss and Jurkat cells. Vertical lines indicate the injection of galactose, glucose or a combination of the two and oligomycin, respectively. Data from primary CD4+ T cells represent the average from four plates with one donor per plate. Data for CEM-ss and Jurkat cells are the average of three plates. Error bars represent the standard deviation and asterisks indicate p-values from ANOVA with Tukey post-test relating to the differences between cultures containing galactose only and cultures containing glucose or glucose and galactose: *0.01 < p < 0.05, **0.005 < p < 0.01, ***p < 0.005, data points without an asterisk have non-significant differences.

Having established that primary CD4+ T cells increase their glycolytic rate in response to infection with HIV-1, we sought to manipulate the metabolism of the cell to determine whether the glycolytic metabolism impacts on the fate of the infected cell and the viral replication cycle. This can be achieved by providing cells with the alternative carbohydrate galactose instead of glucose. Lymphocytes grown in galactose rely mostly on oxidative metabolism [16]. Galactose is unable to enter the glycolytic pathway directly but it can support cell proliferation through entering the pentose phosphate pathway [31]. Small amounts of galactose-derived carbon can enter glycolysis via the Leloir pathway [32] but this process is inefficient. We demonstrated this by monitoring the ECAR and OCR of uninfected primary CD4+ T cells upon the provision of either galactose or glucose, with subsequent addition of oligomycin. Indeed, provision of galactose failed to raise the ECAR to the extent that glucose did (Figure 2A) in the absence or presence of oligomycin. Furthermore, provision of galactose did not suppress the OCR (Figure 2B). When cells were provided with galactose as well as glucose the results were identical to those cultures treated with glucose only, demonstrating that galactose does not interfere with the ability of cells to metabolise available glucose. We performed the same analysis with Jurkat and CEM-ss cells (Figure 2A and B), which demonstrated that the cell lines are likewise unable to efficiently use galactose as a substrate for glycolysis. Interestingly, the Crabtree effect is essentially absent from CEM-ss cells. Infected primary CD4+ T cells, as well as Jurkat and CEM-ss cells, showed no consistent differences in ECAR and OCR upon provision of galactose compared with uninfected cells (Additional file 4), suggesting that oxidative metabolism of T cells is not grossly altered upon infection with HIV-1.

**Figure 3:**
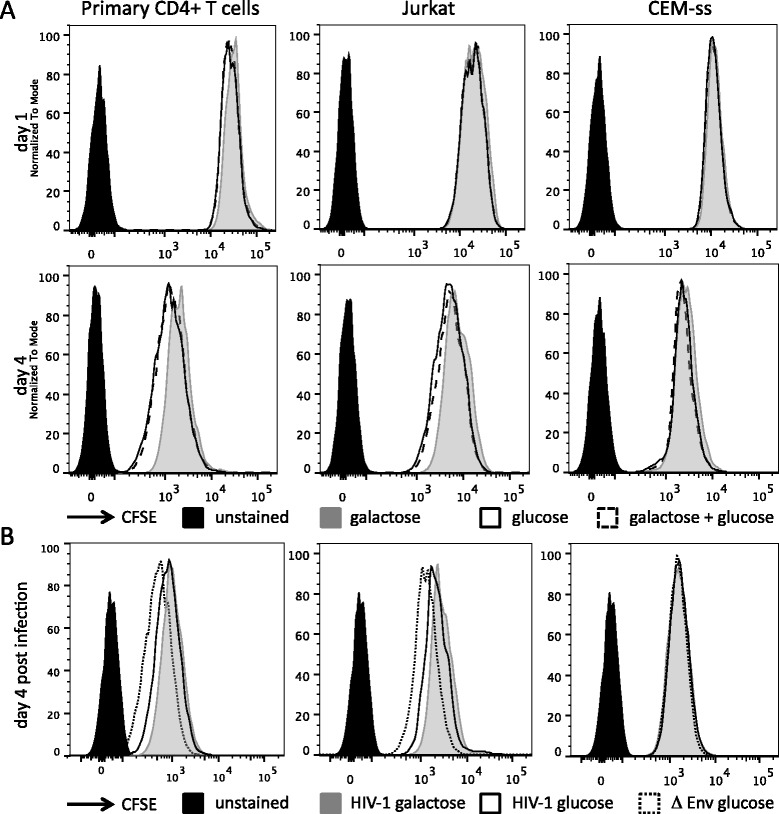
**Cell proliferation of HIV-1 infected and uninfected T cells in DMEM with galactose or glucose. A**. Cell proliferation of T cells as determined by CFSE dilution. Primary CD4+ T cells (donor 3) or T cells lines were stained with CFSE and cultured for 4 days in DMEM with 1 g/L of either galactose, glucose or a combination of both. At day 1 and day 4 cells were harvested and analysed by flow cytometry. **B**. Proliferation of primary CD4+ T cells, Jurkat and CEM-ss cells infected with HIV-1 NL4.3 (HIV-1) or non-infectious Env-deleted HIV-1 NL4.3 (Δ Env) in DMEM containing 1 g/L galactose or glucose. Data for HIV-1 infected cells represent the population of cells that were gated to contain p24^Gag^-expressing cells only.

In order to further characterise the impact of culturing primary CD4+ T cells in galactose versus glucose, we performed CFSE dilution experiments to monitor cell proliferation. Primary CD4+ T cells are able to proliferate when provided with galactose as evidenced by gradual dilution of the intracellular CFSE signal over a period of four days (Figure 3A and Additional file 5). As was previously observed with murine CD4+ T cells [16], the human primary CD4+ T cells showed a slight delay in proliferation rate when grown in media containing galactose compared with glucose. This difference in proliferation rate was not observed with CEM-ss cells (Figure 3A). Throughout these experiments, cultures provided with galactose as well as glucose behaved identically to those provided with glucose only.

We also monitored the proliferation of HIV-1 NL4.3 infected cells and observed that proliferation of infected primary CD4+ T cells cultured with glucose or galactose was retarded compared with uninfected cells cultured with glucose. The effect of culturing infected primary CD4+ T cells in galactose on proliferation was variable, with two donors presenting delayed proliferation compared with infected cells provided with glucose whereas the cells from donor 1 did not (Figure 3B and Additional file 5). A difference in proliferation between infected cells in media containing galactose or glucose was completely absent with CEM-ss cells (Figure 3B).

### HIV-1 infected primary CD4+ T cells have a survival advantage when cultured in galactose compared with glucose

**Figure 4:**
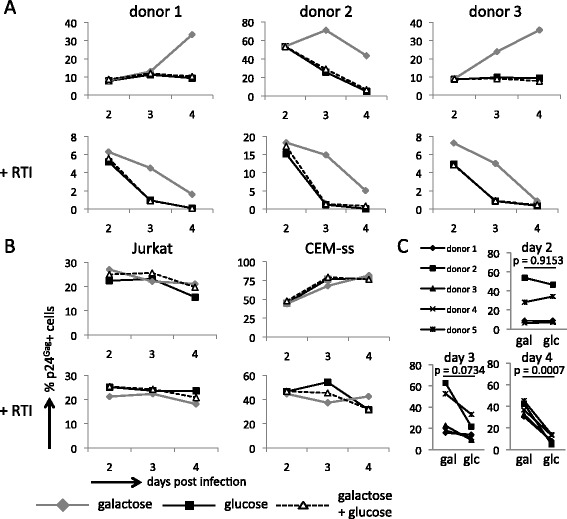
**Cells infected with HIV-1 NL4.3 have a survival advantage when cultured in media containing galactose compared with glucose. A**. Primary CD4+ T cells were infected with HIV-1 NL4.3 in RPMI with IL-2. After 24 hours cells were washed and seeded into DMEM containing galactose, glucose or a combination of the two in the absence or presence of reverse transcriptase inhibitors (RTI). Cells were harvested at 24 hour intervals and analysed for intracellular HIV-1 p24^Gag^ expression by flow cytometry. Data are shown as the percentage of p24^Gag^ positive cells. **B**. The percentage of p24^Gag^ positive Jurkat and CEM-ss cells cultured in DMEM containing galactose, glucose or a combination of the two in the absence or presence of reverse transcriptase inhibitors (RTI). **C**. Comparison of the percentage of p24^Gag^ positive primary CD4+ T cells in cultures containing galactose (gal) or glucose (glc) from five donors for each day in the time course experiment, with the associated p-values as calculated with a paired t-test.

We next monitored the impact of providing cells with galactose or glucose on the course of infection. Jurkat, CEM-ss and primary CD4+ T cells were infected with HIV-1 NL4.3 for 24 hours in standard RPMI media, after which they were washed and split into cultures containing galactose, glucose or a mixture of both. This strategy ensured that the cells experienced an identical level of infection at the start of the experiment and that subsequent differences in the course of the infection would be strictly due to the nature of the carbohydrate provided. Samples were then taken each subsequent day for three days and assessed for the proportion of HIV-1 infected cells as determined by intracellular staining for the viral p24^Gag^ antigen. This revealed that the primary CD4+ T cells from cultures provided with galactose contained a higher proportion of HIV-1 NL4.3 infected cells than cells from cultures containing glucose only or a combination of glucose and galactose (Figure 4A and Additional file 6). This difference reached statistical significance on day four post infection (Figure 4C). The cultures were also split into samples that additionally received reverse transcriptase inhibitors (RTI) in order to monitor differences occurring after one round of infection only (Figure 4A and Additional file 6: +RTI). In the presence of RTI a higher proportion of infected cells in cultures containing galactose compared with glucose was maintained. This implies that, as the initial level of infection was identical, the difference in the amount of cells that express viral p24^Gag^ antigen is due to a difference in the rate at which the infected cells succumb to cell death in media containing galactose or glucose. Specifically, HIV-1 infected primary CD4+ T cells grown in cultures containing galactose appear to have a survival advantage over infected cells grown in glucose. Strikingly, no difference in the level of infection due to the nature of the carbohydrate was observed with Jurkat and CEM-ss cells, either in the presence or absence of RTI (Figure 4B).

As we had previously observed that the nature of the carbohydrate does impact on the proliferation rate of primary cells, we considered that the differential proportion of HIV-1 infected cells could be affected by outgrowth of uninfected cells. This could particularly affect cultures containing glucose as this carbohydrate supports faster proliferation. Thus, we sought to determine if the differences between cultures containing galactose or glucose might be caused by an outgrowth of uninfected cells in cultures containing glucose, thereby reducing the relative proportion of infected cells. We therefore determined the actual number of cells in each sample using CountBright beads (Additional file 7). When this adjustment was applied, counts of HIV-1 infected primary CD4+ T cells remained higher in cultures containing galactose than in cultures containing glucose.

We performed the same experiment with the YU2 isolate and again observed that cultures containing galactose had a higher proportion of infected cells compared with cultures containing glucose (Additional file 8). We note that, compared with NL4.3, the magnitude of this effect was lower in cells infected with YU2. The difference in the corrected amount of p24^Gag^ positive cells in cultures containing galactose or glucose was readily discernable in the presence of RTI for primary CD4+ T cells infected with YU2. As with NL4.3, Jurkat and CEM-ss cells infected with YU2 showed no difference in the amount of p24^Gag^ positive cells in response to the carbohydrate provided.

### Caspase 3 activation is increased in primary CD4+ T cells cultured in glucose compared with galactose

To investigate the nature of the survival advantage that HIV-1 infected cells experience in media containing galactose, we measured the expression of several cellular proteins that are known to impact on cell survival or apoptosis. In particular, we assessed the intracellular expression of the pro-apoptotic activated caspase 3, the regulator of cell survival pathways phosphorylated AKT1 (p-AKT1) and the anti-apoptotic Bcl2 by flow cytometry. These cellular factors have all previously been implicated in controlling cell death in HIV-1 infections [33-40].

**Figure 5:**
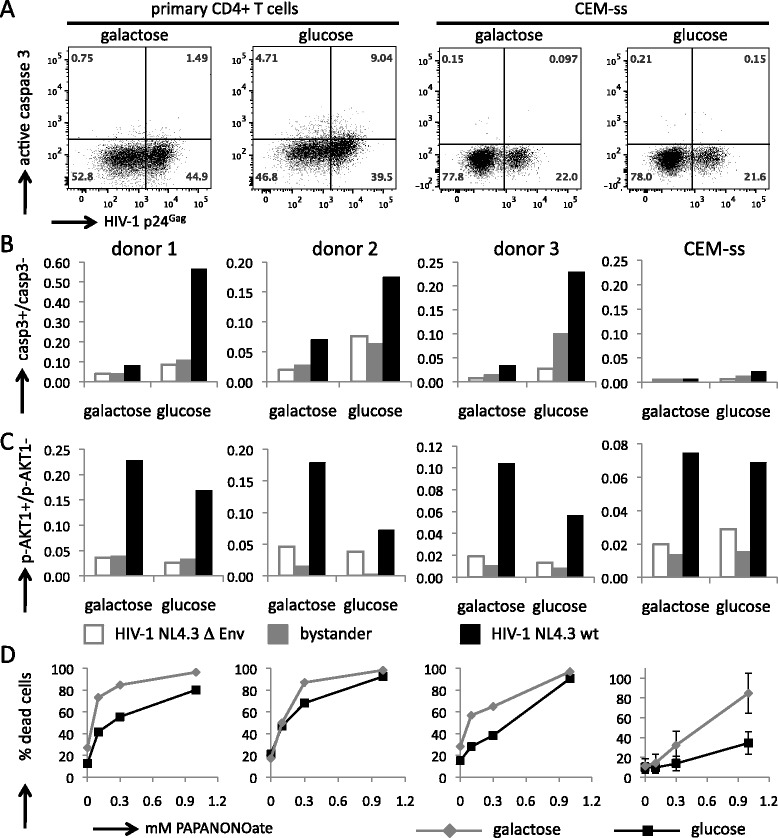
**A greater proportion of cells infected with HIV-1 NL4.3 express activated caspase 3 when cultured in media containing glucose compared with galactose. A**. Scatter plots demonstrating the gating of p24^Gag^ and activated caspase 3 positive populations of HIV-1 infected primary CD4+ T cells and CEM-ss cells. The data shown correspond to day 2 post infection, which represents 1 day of culturing in media containing 1 g/L galactose or glucose. **B**. The ratio of activated caspase 3 positive to negative cells after 24 hours of culturing in media containing galactose or glucose for primary CD4+ T cells from 3 different donors and CEM-ss cells. **C**. The ratio of phosphorylated AKT1 (p-AKT1) positive to negative cells after 24 hours of culturing in media containing galactose or glucose for primary CD4+ T cells from 3 different donors and CEM-ss cells. **D**. Percentage of dead cells as determined by staining with propidium iodide of primary CD4+ T cells from three different donors and CEM-ss cells exposed to increasing concentrations of PAPANONOate for 24 hours in media containing galactose or glucose. Experiments with primary cells were performed once for each donor. Experiments with CEM-ss cells were performed in triplicate with the error bars indicating the standard deviation.

We observed an increased proportion of cells expressing activated caspase 3 in infected primary CD4+ T cells that were grown in glucose compared with infected cells grown in galactose (Figure 5A and B). We note that a slight enhancement of the number of cells expressing activated caspase 3 was also apparent in uninfected and bystander cells that were grown in glucose compared with galactose, suggesting that caspase 3 activation may be more generally linked to glycolytic metabolism of the cell. However, active caspase 3 in uninfected and bystander cells grown in glucose was markedly lower than in infected cells, demonstrating that activation of caspase 3 contributes to apoptotic cell death in HIV-1 infected cells. The number of cells expressing activated caspase 3 was also increased in primary CD4+ T cells when infected with HIV-1 YU2 and cultured in the presence of glucose compared with galactose (Additional file 9). Thus, we consistently observed that the number of cells expressing activated caspase 3 was low in cultures of primary CD4+ T cells that contained galactose, which correlates with their enhanced cell survival (Figure 4 and Additional files 6, 7 and 8). In contrast, activation of caspase 3 in infected CEM-ss (Figure 5 and Additional file 9) and Jurkat (not shown) cells was roughly an order of magnitude lower than in the primary CD4+ T cells with either carbohydrate. Consistent with a glycolysis-dependent mechanism of caspase 3 activation, we observed that primary CD4+ T cells infected with the reporter virus NL4.3-GFP contained greater populations of infected as well as uninfected cells that stained positive with annexin V in cultures containing glucose compared with galactose (Additional file 10). Again, this difference was not observed with CEM-ss cells.

The protein kinase AKT1 acts as a master regulator of cell fate by controlling processes such as metabolism, proliferation and apoptosis, and its kinase activity is activated by phosphorylation [41,42]. We observed that p-AKT1 is more abundant in HIV-1 infected CEM-ss and primary CD4+ T cells, regardless of the carbohydrate provided in the cultures. A slight increase of p-AKT1 in NL4.3 infected cells cultured in galactose compared with glucose was consistently observed in primary CD4+ T cells but not in CEM-ss cells (Figure 5C). No differences in the levels of p-AKT1 in response to the carbohydrate provided was observed when primary CD4+ T cells were infected with the HIV-1 YU2 isolate (Additional file 9). Thus, although we cannot rule out that activation of AKT1 by phosphorylation may contribute to the survival advantage that HIV-1 infected primary CD4+ T cells experience in cultures containing galactose, our current data do not support a major role for AKT1 in regulating cell survival in this context. Likewise, investigation of the intracellular expression of Bcl2 by flow cytometry did not show a dependency on the carbohydrate substrate (not shown). We did observe that Bcl2 expression was increased in HIV-1 infected cells as is consistent with a recent report [40].

It has recently been demonstrated that the translation of the cytokines IL-2 and IFNγ is dependent on glycolytic metabolism in murine CD4+ T cells [16]. IL-2 in particular is known to affect T cell proliferation and its translational regulation may therefore impact on the survival and expansion of the different cell populations in our experiments. In addition, the provision of galactose or glucose in the culture media may skew the proportion of effector T cell subsets in our experiments through glycolysis-driven control mechanisms. We therefore determined the intracellular expression levels of IL-2, IL-4, IFNγ and TNFα in the infected and uninfected CD4+ T cell populations. These analyses indicated that there was no effect of the choice of carbohydrate on the production of these cytokines or the composition of Th1 and Th2 compartments during the course of our experiments (Additional file 11). It is important to note that in our experiments primary CD4+ T cells were activated and expanded in standard RPMI containing glucose for up to five days prior to infection with HIV-1 and subsequent provision of either glucose or galactose. Therefore, the initial burst of cytokine production after activation would not have been subject to the effects mediated by the provision of glucose or galactose in the culture media [16]. We conclude therefore that, once activated, the differential survival of HIV-1 infected primary CD4+ T cells in cultures containing glucose or galactose is not due to expression and autocrine action of IL-2, IL-4, IFNγ or TNFα.

Because cells grown in galactose are mostly dependent on oxidative phosphorylation for their metabolic needs, we considered that this might impact on their sensitivity to oxidative stress. The generation of reactive oxygen species (ROS) in HIV-1 infected cells has been reported to affect viral gene expression as well as HIV-induced cell death [43-46]. In addition, it has been shown that glucose protects epithelial cells and lymphocytes from oxidative damage through the generation of ROS-scavenging species such as lactate and pyruvate as well as by the maintenance of reducing equivalents [47-49]. In order to assess if oxidative damage may impact on the differential survival of CD4+ T cells in media containing glucose or galactose, we subjected the cells to oxidative damage by the nitric oxide (NO) donor molecule PAPANONOate and monitored cell death by staining with propidium iodide (Figure 5D). Cells that were grown in glucose consistently showed resistance to NO-induced cell death compared with cells grown in galactose, and this was true of primary CD4+ T cells, CEM-ss (Figure 5D) and Jurkat cells (not shown). As glucose therefore confers resistance to oxidative stress, we conclude that the increased survival of HIV-1 infected cells grown in galactose is not attributable to mechanisms involving differences in oxidative damage.

### Glucose supports virion production during the HIV-1 life cycle

We next set out to determine what stages of HIV-1 replication are affected by the nature of carbohydrate provided to target cells. To investigate the early stages of HIV-1 replication, we seeded cells in media containing either glucose or galactose and subsequently added equal amounts of virus. After 24 hours, cells were harvested and assessed for intracellular HIV-1 p24^Gag^ levels. At 24 hours only a single round of HIV-1 infection would have been completed and the amount of p24^Gag^ expressed in cells would therefore be the result of successful entry, integration and viral gene-expression. We observed a small but reproducible reduction of cells that stained positive for p24^Gag^ in cultures containing galactose compared with glucose (Additional file 12). This indicates that early steps of virus replication from entry to gene-expression are moderately affected by the ability of the target cell to perform glycolysis at the time of infection.

**Figure 6:**
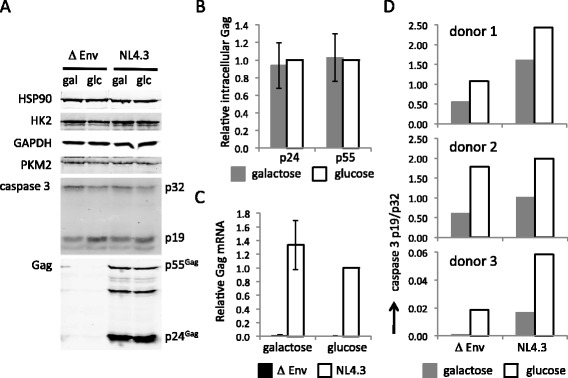
**Expression of glycolytic enzymes and HIV-1 Gag in primary CD4+ T cells is not affected by culturing of cells in media containing galactose or glucose. A**. Western blot showing the expression of heat shock protein 90 (HSP90), hexokinase 2 (HK2), glyceraldehyde-3-phosphate dehydrogenase (GAPDH), pyruvate kinase M2 (PKM2), pro-caspase 3 (p32), activated caspase 3 (p19) and HIV-1 Gag (p24^Gag^ and p55^Gag^) in HIV-1 infected (NL4.3) and uninfected (Δ Env) primary CD4+ T cells for one representative donor (donor 2). Lanes corresponding to cultures containing galactose are indicated with gal and lanes corresponding to cultures containing glucose are indicated with glc. **B**. The relative amount of p24^Gag^ and p55^Gag^ HIV-1 in primary CD4+ T cells from cultures containing galactose or glucose as quantified from western blots. p24^Gag^ and p55^Gag^ were divided by the amount of HSP90 in each sample and normalised to the amount present in cultures containing glucose. The data are the average of a total of five western blots from independent infections of primary CD4+ T cells from three different donors with NL4.3. Errors bars represent the standard deviation. **C**. The amount of HIV-1 unspliced mRNA relative to β-actin mRNA in primary CD4+ T cells infected with NL4.3 or NL4.3 Δ Env as determined by qPCR. Data are normalised to the amount of HIV-1 Gag mRNA in cultures containing glucose and are the average of five independent infections of primary CD4+ T cells from three different donors. Error bars indicate the standard deviation. **D**. The ratio of activated caspase 3 p19 to pro-caspase 3 p32 in HIV-1 infected (NL4.3) and uninfected (∆ Env) primary CD4+ T cells from cultures containing galactose or glucose as quantified from western blots.

To investigate effects of glycolysis on late stages of virus replication, cells were infected in RPMI for 24 hours and split into cultures containing galactose or glucose as before. Western blotting of the cell lysates revealed that expression of the HIV-1 Gag proteins p55^Gag^ and p24^Gag^ was unaffected by culturing the cells in the presence of glucose or galactose for a further 24 hours (Figure 6A and B). Likewise, the intracellular level of HIV-1 Gag mRNA was equivalent in media containing galactose or glucose (Figure 6C). Interestingly, western blot analysis also revealed that expression of glycolytic enzymes hexokinase 2 (HK2), glyceraldehyde-3-phosphate dehydrogenase (GAPDH) and pyruvate kinase M2 (PKM2) was unaffected by the identity of the carbohydrate (Figure 6A). In addition, there was no difference in the expression of these glycolytic enzymes in uninfected and infected cells, as judged by western blotting (Figure 6A). We also observed an increase in the levels of activated caspase 3 in cultures containing glucose by western blotting, with the highest proportion of cleaved p19 to uncleaved p32 in HIV-1 NL4.3 infected cells (Figure 6A and D).

**Figure 7:**
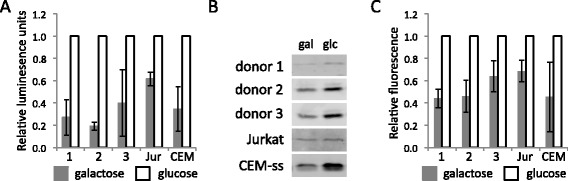
**HIV-1 virion production is dependent on glycolysis. A**. Infectivity of culture supernatants from HIV-1 NL4.3 infected primary CD4+ T cells, Jurkat (Jur) and CEM-ss (CEM) cells that were cultured for 24 hours in media containing 1 g/L glucose or galactose, was determined by β-galactosidase activity of the indicator cell line TZM-bl. **B**. Western blots showing the presence of p24^Gag^ in the supernatant from cultures of HIV-1 NL4.3 infected primary CD4+ T cells , Jurkat and CEM-ss in cultures containing 1 g/L galactose (gal) or glucose (glc). **C**. Quantified data from western blotting of p24^Gag^ shown in (B). All experiments were performed in triplicate and are shown as the average normalised to the infectivity of cultures containing glucose. Error bars represent the standard deviation.

Finally we assessed whether the carbohydrate metabolism of HIV-1 infected cells affected the production of viral particles. Cells were infected in RPMI and subsequently cultured in media containing galactose or glucose for 24 hours. The supernatants of these cultures were harvested and assessed for viral infectivity using the indicator cell line TZM-bl and the presence of viral p24^Gag^ protein by western blotting (Figure 7A, B and C). These analyses revealed that virion production in cultures containing galactose was reduced to 20–60% of the amount produced in cultures containing glucose. The results from western blotting mirrored those obtained from the infectivity assay with TZM-bl cells. We observed the same reduction of virion production in cultures containing galactose compared with glucose when cell were infected with the HIV-1 YU2 isolate (not shown). As the presence of intracellular Gag was unaffected by culturing in galactose or glucose (Figure 6A), these data indicate that there is a specific requirement for glycolysis to support HIV-1 virion production. We also performed experiments in which infected cells were split into cultures containing only glucose or a mixture of glucose and 2-deoxyglucose, which is a competitive inhibitor of glycolysis (Additional file 13). The cultures containing 2-deoxyglucose contained less virus in the supernatant as assessed with the TZM-bl assay and western blotting, further supporting the notion that glycolysis is required to support HIV-1 virion production. We also noted that the number of live primary CD4+ T cells in cultures containing 2-deoxyglucose was reduced to approximately half that of the cultures containing glucose, whereas Jurkat and CEM-ss cell viability was hardly affected by 2-deoxyglucose (not shown).

## Discussion

We report an investigation into the dependency of HIV-1 infections on the highly glycolytic metabolism of its main target cells, the activated CD4+ T cells. The elevated state of glycolysis that persists in activated T cells is required to support their effector functions, which place high biosynthetic demands on the cells to produce cytokines. Although it was previously thought that the proliferation of activated T cells is driven mainly by glycolysis, recent work has demonstrated that cell proliferation can be fuelled by oxidative phosphorylation alone, whereas cytokine production is inhibited in the absence of glucose [16]. We therefore asked whether the anabolic metabolism provided by activated T cells undergoing glycolysis might be exploited by HIV-1 to support virus replication. Indeed, we observed that both the fate of the HIV-1 infected cell and HIV-1 virion production are dependent on glycolysis.

It had previously been demonstrated that HIV-1 infected CD4+ T cells contain higher levels of glycolytic intermediates [22]. However, increased levels of metabolites may indicate either a higher rate of production or a lower rate of depletion, and are therefore not indicative of the activity of the pathway in question. Although Hollenbaugh *et al.* demonstrated an increase in glucose uptake in HIV-1 infected cells, intracellular levels of lactic acid were similar to those of uninfected cells. In addition, increased uptake of 2-deoxyglucose in HIV-1 infected H9 cells in culture has previously been reported [50]. Our study complements those observations by demonstrating that there is indeed increased flux through the glycolytic pathway in primary CD4+ T cells upon infection with HIV-1. Extracellular flux measurements in the presence of oligomycin suggested that HIV-1 infected cells could be operating at their maximal glycolytic capacity. We did note a small shift in the median fluorescence intensity of the surface-expressed glucose transporter GLUT1 on HIV-1 infected cells, which may suggest a small increase in the abundance of the transporter in comparison with uninfected cells. However, this would only account for an increase in glycolytic activity if glucose transport were rate limiting to glycolysis in HIV-1 infected primary CD4+ T cells. This remains to be established. In this context, we note that increased expression of GLUT1 in CD4+ T cells from HIV-1 infected individuals has recently been suggested as a marker of T cell activation as well as being prognostic of disease progression [51].

Western blotting of several glycolytic enzymes suggested that increased glycolytic flux proceeds without altering the expression levels of these proteins in HIV-1 infected primary CD4+ T cells. The HIV-1 mediated increase of glycolysis may also be achieved by several possible mechanisms including assembly of higher order complexes, post-translational modification or allosteric regulation of glycolytic enzymes. For example, it was recently reported that the binding of the hepatitis C virus protein NS5A increased the enzymatic activity of HK2, leading to a general increase in glucose consumption and lactic acid production [52]. On the other hand, infection of Vero cells with mayaro virus was shown to increase the activity of phosphofructokinase (PFK) [53]. Cells infected with herpes simplex virus were recently shown to have increased glucose uptake and lactate efflux that correlated with upregulation and phosphorylation of PFK [54]. In cancers, glycolytic flux is responsive to the assembly of PKM2 into dimers or tetramers, which determines the fate of glucose-derived carbon towards biosynthesis or oxidative phosphorylation, respectively [55]. Exactly how HIV-1 exerts control over glycolysis remains to be determined.

We found no evidence to suggest that oxidative phosphorylation was affected in HIV-1 infected cells, which is also in agreement with generally unaffected levels of TCA cycle intermediates [22]. This suggests therefore that HIV-1 replication has a specific requirement for resources that derive from glycolysis. We only observed an increase in glycolytic flux in primary CD4+ T cells after infection with HIV-1 but not in the T cell lines Jurkat and CEM-ss. Both these cell lines are derived from leukemic patients and it is well established that a hallmark of transformed cells is the Warburg effect, which is characterised by increased glycolytic activity despite the presence of sufficient oxygen to support oxidative phosphorylation [56]. Our results suggest that in these cell lines glycolysis is de-regulated to the extent that HIV-1 exerts no further control over the upregulation of glycolysis.

By monitoring the number of cells that expressed HIV-1 p24^Gag^ in media containing galactose or glucose we noted that a higher proportion of infected primary CD4+ T cells was maintained in those cultures containing galactose. By the addition of reverse transcriptase inhibitors, we showed that this was independent of subsequent rounds of infection and therefore suggested that galactose provides infected cells with a survival advantage over glucose. Indeed, a greater proportion of cells expressing activated caspase 3 was observed in the infected primary CD4+ T cells cultured in glucose. We noted that an increased proportion of cells expressing activated caspase 3 was not only observed in infected cells but also in uninfected cells cultured in glucose, suggesting a more general connection between glycolytic metabolism and apoptotic cell death in activated CD4+ T cells. This was furthermore reflected in experiments that revealed a greater proportion of infected as well as uninfected cells that stained positive with annexin V in cultures containing glucose compared with galactose. Increased sensitivity of activated T cells to apoptosis under conditions that favour glycolysis has been reported [57] as well as the requirement for glycolytic ATP production for apoptosis of Jurkat cells [58]. It was recently reported that IL-2-dependent activation-induced cell death of murine effector T cells is mediated by activated caspase 3 and this was correlated with increased glycolytic activity [59]. In addition, glycolysis-dependent activation of caspase 3 was recently reported to limit inflammatory cytokine production by a negative feedback mechanism in macrophages after sustained activation [60]. In conjunction with our present work, these studies point to a central role of caspase 3 in mediating metabolism-dependent contraction of immune responses as well as pathogen-induced cell death. We emphasise that we are not advocating a strict caspase 3-dependent mechanism of HIV-1 induced apoptotic cell death. Necrosis [61] and necroptosis [62] have been observed in HIV-1 infected cells in addition to several possible modes of apoptosis [33-36,39,61] and non-productively infected cells were recently shown to die by caspase 1-mediated pyroptosis [63].

In addition to an effect of cellular metabolism on HIV-1 induced cell death, we also observed that virus production is more efficient in cells provided with glucose compared with galactose. This is not unexpected, as glycolysis generates intermediates that support biosynthesis of amino acids, lipids, nucleotides, glycolipids and glycoproteins. In fact, it is likely that our experiments comparing cultures containing galactose or glucose have underestimated the dependency of HIV-1 virion production on glycolysis to some extent. Galactose can be metabolised via the Leloir pathway to produce UDP-galactose that can support nucleotide sugar metabolism or be used to generate glucose-1-phosphate that can enter glycolysis, albeit inefficiently. Nevertheless, we observed that virus production in cultures containing galactose was reduced to between 20% and 60% of the amount produced in cultures containing glucose. Likewise, the addition of 2-deoxyglucose to infected cell cultures reduced the viral output severely, but there is also some cellular toxicity associated with 2-deoxyglucose. To our knowledge, this is the first time that a dependence of HIV-1 virion production on glycolysis has been demonstrated.

There are several possibilities by which glucose would be required for the synthesis of biomolecules that make up the virion. For instance, there is evidence that lipid metabolism is modulated in HIV-1 infected cells to support the formation of lipid microdomains that are the preferred assembly sites for HIV-1 virions [64,65]. This may pose an increased demand on the production of precursor metabolites for lipid metabolism that are derived from glycolysis, such as acetyl-CoA. In addition, the gp120 subunit of the viral Env protein is one of the most heavily glycosylated proteins found in nature and its synthesis may well require an increase in sugar molecules that are targeted into protein glycosylation pathways. Alternatively, there may be host factors that are required for HIV-1 particle production whose expression or activity depends on glycolytic metabolism. For example, the translation of IFNγ and IL-2 mRNA is repressed by the binding of GAPDH to the 3′ UTR when it is not engaged in glycolytic catalysis [16]. It is conceivable that other host protein that support HIV-1 replication may be subject to similar control mechanisms. Our observation that HIV-1 Gag protein and its mRNA levels are similar in cultures containing galactose or glucose indicates that these components of the HIV-1 virion are not subject to translational control by GAPDH. Interestingly, we observed that virus production was dependent on the provision of glucose in cell lines as well as primary cells. Together, these results indicate that the process of virion assembly has a metabolic requirement that is conserved in primary CD4+ T cells and T cell lines.

## Conclusions

We have demonstrated that the infection of primary CD4+ T cells, but not T cell lines, with HIV-1 results in an increased glycolytic flux. Furthermore, we have shown that the engagement of glycolysis in infected primary CD4+ T cells makes them more susceptible to virus-mediated cell death while at the same time supporting virion production. Increased apoptosis and activation of caspase 3 was shown to correlate with glycolytic activity of primary CD4+ T cells, both in the presence and absence of HIV-1.

## Methods

### Extracellular flux measurements

Oxygen consumption and extracellular acidification rates, OCR and ECAR respectively, were measured on an XF24 apparatus from Seahorse Biosciences. All extracellular flux experiments were carried out in Dulbecco Modified Eagle Medium (DMEM) reconstituted from powder formulation (Sigma) supplemented with 10 mM pyruvate and 1 mM glutamine but without sodium bicarbonate, phenol red or serum. The DMEM was prepared on the day of the experiment and adjusted to a pH between 7.2 and 7.4 at 37°C, and subsequently filter-sterilised. Cells were washed once in DMEM and seeded at a density of 1.5 to 2.0 × 10^5^ per well in XF24 culture plates that were coated with Cell-Tak (BD biosciences) or poly-D-Lysine (Sigma) and centrifuged for 5 minutes at 135 g at medium acceleration. No differences in results were observed when using Cell-Tak or poly-D-Lysine. After centrifugation, the cell plate was directly loaded into the instrument. Glucose and galactose were injected to a final concentration of 1 g/L and oligomycin to a final concentration of 1 μM. HIV-1 and uninfected cells were assayed 48 hours after infection, HIV-1 infected cells stained at least 70% positive for viral p24^Gag^ antigen as determined by flow cytometry. In experiments comparing addition of glucose, galactose and a combination of the two, 7 wells were designated for each condition with 3 wells dedicated to background detection. In experiments comparing the addition of glucose and galactose, 10 wells were dedicated to each condition and 4 for background detection. All results were normalised to the baseline OCR and ECAR values recorded prior to the injection of substrate. Data are represented as the average with error bars indicating the standard deviation.

### Cell culture

Peripheral blood mononuclear cells (PBMCs) were isolated from whole blood by density centrifugation using Lymphoprep (StemCell Technologies). CD4+ T cells were isolated by magnetic activated cell sorting (MACS) by negative selection (Miltenyi Biotec). The purity of the isolated CD4+ T cell population was determined by flow cytometry and was typically ≥95%. Cells were frozen in 30% foetal bovine serum (FBS)/10% dimethyl-sulfoxide (DMSO) until needed; upon which aliquots were thawed, activated with CD3/CD28 T-cell activator Dynabeads (12.5 μl per 10^6^ cells) (Invitrogen) and cultured in RPMI-1640 containing 10% FBS, 1% penicillin/streptomycin (P/S) and 15 international units/ml (IU/ml) IL-2 for 4 days to expand (all Invitrogen). CD4+ T cells lines, Jurkat and CEM-ss were maintained in RPMI-1640 containing 10% FBS and 1% P/S. Human Endothelial Kidney 293 T (HEK 293 T) and TZM-bl cells [66,67] were maintained in DMEM (Invitrogen) supplemented with 10% FBS and 1% P/S. DMEM containing 1 g/L galactose or glucose carbohydrate was made up using the powder formulation of DMEM (Sigma) that lacks glucose, pyruvate, sodium bicarbonate, glutamine and phenol red: these components (Sigma) were added to obtain the standard DMEM formulation. In addition, 10% dialysed FBS (Sigma), 1% P/S and 15 IU/ml IL-2 (both Invitrogen) were added and media passed through 0.2 μm filter to sterilise.

### Virus production and infection

HIV-1 YU2 [68], NL4.3-nef-IRES-GFP (NL4.3-GFP) [69,70], NLRX-IRES (NL4.3-DSredX) [71], NL4.3 [72] and negative control Env-deleted virus (NL4.3 Δ Env [73,74]) virus were produced by transfection of the molecular clones with PEI (Polysciences) into HEK 293 T cells. YU2 viruses were pseudotyped with the vesicular stomatitis virus glycoprotein (VSV-G) by co-transfection of a VSV-G expression vector. Supernatant from transfected cells was purified by filtering through a 0.45 μm mixed cellulose membrane (Millipore) and subsequent ultra-centrifugation over a 20% sucrose cushion for 75 minutes at 28,000 rpm. Virus pellets were resuspended in glucose-free DMEM and virus infectivity was then determined by titration on CEM-ss and Jurkat cells with subsequent intracellular p24^Gag^ staining and flow cytometry analysis. The volume of viral stock needed to achieve the desired percentage of infected cells was then calculated for each experiment. For infection experiments, primary CD4+ T cells were re-activated with anti-CD3/CD28 beads and infected in RPMI + IL-2. Jurkat and CEM-ss cells were infected in RPMI only. Following 24 hours incubation at 37°C, cells were washed with PBS and seeded into DMEM, containing IL-2 and 1 g/L galactose, glucose or a combination of the two. Cells were infected and cultured in the presence or absence of reverse transcriptase inhibitors (RTI) Zidovudine and Lamivudine (10 μM each: obtained through the NIH AIDS Reagent Program, Division of AIDS, NIAID, NIH).

### Flow cytometry

Infected cells were washed in PBS and treated with TrypLE (Sigma) to remove membrane bound HIV-1, before being permeabilised and fixed with Cytofix/Cytoperm (BD Biosciences). Cells were then incubated with either p24^Gag^ antibody only or in combination with Bcl2 or caspase 3 antibodies in Permwash (BD Biosciences). p-AKT1 was detected by washing cells in PBS, treating with TrypLE, fixing in 4% paraformaldehyde and incubating with cold Perm Buffer III (BD Biosciences) to capture the protein in its phosphorylated form. Cells were incubated with anti-p24^Gag^ and p-AKT1 antibodies in FACS buffer (PBS containing 2% FBS and 1% P/S). For detection of intracellular cytokines (IL-2, IL-4, TNFα and IFNγ) Brefeldin A (Biolegend) was added at 1:1000 to primary CD4+ T cells and incubated for 6 hours at 37°C. Cells were then washed in PBS and incubated with Live/Dead Fixable Dead Cell Stain Kit violet (Invitrogen) for 30 minutes in the dark at room temperature (RT). Next cells were washed, treated with TrypLE, fixed and permeabilised with Cytofix/Cytoperm (BD Biosciences) and stained with anti-p24^Gag^ antibody plus either anti-TNFα; IL-2 and IFNγ; or IL-4 and IFNγ. GLUT1 expression was detected in cells infected with NL4.3-GFP virus after permeabilisation and fixing with Cytofix/Cytoperm and incubated with mouse anti-human GLUT1 antibody at RT for 30 minutes. After washing with FACS buffer cells were incubated with anti-mouse-PE secondary antibody at RT for 30 minutes in the dark. Cells were washed in FACS buffer 3 times then resuspended in FACS buffer before being acquired on a FACSCantoII and analysed on FlowJo software (Treestar). Where required, CountBright Absolute Counting Beads (Invitrogen) were vortexed for 30 seconds and 50 μl added to each sample prior to acquisition. The following antibodies were used at concentrations indicated in the manufactures’ instructions: anti-p24^Gag^-FITC and anti-p24^Gag^-RD1 KC57-RD1 (Beckman Coulter), anti-Bcl2-PE-CF594 clone 100, anti-activated caspase 3-FITC clone C92-605, anti-p-AKT1-pacific blue clone M89-61, anti-TNFα-APC clone 6401.1111, anti-IL-2-FITC clone 5344.11, anti-IL-4-PE clone 3010.211, anti-IFNγ-APC clone 25723.11, anti-CD4-PE RPA-T4 (all from BD Biosciences), anti-GLUT-1 Ab40084 (Abcam), GLUT1-RBD-GFP (Metafora Biosystems) and anti-mouse-PE secondary antibody F0102B (R&D Systems).

### Cell proliferation assay

Cells were washed twice in PBS (400 g/5mins) and resuspended in 1 μM CFSE for 10 minutes at RT in the dark (Invitrogen). The staining was quenched with sterile FACS buffer for 2 minutes and then cells were washed in fresh FACS buffer. Cells were seeded into media containing glucose, galactose or a combination of the two and left to grow for the indicated times with subsequent analysis by flow cytometry. For analysis of infected cells, staining was performed in the same manner and cells were subsequently seeded into RPMI and given an inoculum of virus. After 24 hours, the cells were washed and split into DMEM containing glucose or galactose. At the indicated time, cells were analysed by flow cytometry: cells were gated for p24^Gag^ expression to obtain the CFSE signal for the HIV-1 infected population.

### Cell death by oxidative damage

Uninfected cells were seeded in DMEM with galactose or glucose and incubated for 24 hours at 37°C. PAPANONOate (Cayman chemical) was added at the indicated concentrations from a 30 mM stock solution in DMSO and the cells were incubated at 37°C for a further 24 hours. Cells were then washed in PBS and stained with propidium iodide (PI) as per the Vybrant Apoptosis Assay Kit instructions (Invitrogen) and analysed by flow cytometry.

### Western blotting

Cell and supernatant samples were separated by SDS-PAGE on an 11% SDS gel at 27 mA for 1 hour. Proteins were then transferred onto nitrocellulose membranes at 16 V over night and blocked for 1 hour in 1% skimmed milk in 0.1% Tween/PBS. Membranes were blotted with primary antibodies for 1 hour at room temperature and washed in 0.1% Tween/PBS. Subsequently, membranes were blotted with secondary conjugated antibody for 1 hour at room temperature and washed again before being visualised on a LI-COR Quantitative Imager. The following antibodies were used according to the manufactures’ instructions: anti-HSP90 sc7947 (Santa Cruz), anti-PKM2 ab38237 (Abcam), anti-HK2 ab104830 (Abcam), anti-GAPDH ab8245 (Abcam), anti-caspase-3 9668S (Cell Signaling), 800CW goat anti-rabbit 926–32211 (LI-COR) and 680RD goat anti-mouse 926–68070 (LI-COR). HIV-1 Gag was detected with supernatant from the mouse hybridoma 183-H12-5C, which was a kind gift from Prof. M. Malim.

### Infectivity assay

TZM-bl cells were plated at 1.5 × 10^5^ per well in a 24-well plate and incubated at 37°C O/N. The next day, 100 μl of supernatant from infected cultures was added to each well. After 24 hours incubation at 37°C, media was aspirated off and 100 μl of Galacto-Star Lysis solution (Applied Biosciences) added to each well. Galacto-Star Reporter Assay Kit for the detection of β-galactosidase activity was then used to determine the infectivity of the lysate according to the manufacturers’ instructions. The value from negative control wells that received no supernatant was subtracted from all experimental values to account for any autoluminesence present in TZM-bl cells.

### Quantitative real-time PCR

RNA was extracted using the Qiagen miRNeasy Mini kit according to the manufacturer’s instructions. cDNA was synthesised using High Capacity cDNA Reverse Transcription Kit (Applied Biosystems) according to the manufacturer’s instructions. qPCR reactions were performed in triplicate, using TaqMan Universal PCR master mix (Applied Biosystems) with 900 nM of each primer and 250 nM probe. After 10 min at 95°C, reactions were cycled through 15 sec at 95°C followed by 1 min at 60°C for 40 cycles. To detect HIV-1 genomic RNA the primers used were GagupNL4.3 (AGGCTGTAGACAAATACTGGG) and GagdownNL4.3 (TTTGATGCACACAATAGAGGA) with the probe GagProbegRNA (FAM-CCCTTCAGACAGGATCAGAAGAACTTAGATCA-TAM) (all from Eurofins). As a cellular control β-actin mRNA was amplified (Life technologies), which all data were normalised against.

### Statistics

Graphpad Prism software was used to perform paired t-test, unpaired t-test and ANOVA with Tukey post-test to calculate p-values as indicated.

## Additional files

Additional file 1:
**Viral burden in infected primary CD4+ T cells and cells lines that were subjected to extracellular acidification and oxygen consumption experiments.** Viral burden was assessed by intracellular staining of viral p24^Gag^ antigen of HIV-1 NL4.3 wt infected cells compared with cells treated with NL4.3 Δ Env. A line on top of the plots indicates the gating boundaries for p24^Gag^-positive cells and the corresponding percentage of infected cells in the culture. Additional file 2:
**Monitoring glycolysis and oxidative phosphorylation in VSV-G-pseudotyped HIV-1 YU2 infected and uninfected CD4+ T cells upon provision of glucose.** A. The extracellular acidification rate (ECAR) and B. oxygen consumption rate (OCR) over time is given as a percentage of the baseline recorded at 41 minutes in the absence of glucose for primary CD4+ T cells from three donors, CEM-ss and Jurkat cells. Vertical lines indicate the injection of glucose and oligomycin, respectively. Data from primary CD4+ T cells represent single plates with 10 wells dedicated to each condition and data for cell lines CEM-ss and Jurkat are the average of three plates. In all cases, infected cell populations were at least 70% positive for HIV-1 p24^Gag^ as determined by flow cytometry. Error bars represent the standard deviation and asterisks indicate p-values from an unpaired t-test: *0.01 < p < 0.05, **0.005 < p < 0.01, ***p < 0.005, data points without an asterisk have non-significant differences. Additional file 3:
**Intracellular and surface GLUT1 expression in infection of primary CD4+ T cells infected with HIV-1 NL4.3-GFP/DSredX.** Flow cytometry analysis of GLUT1 expression in primary CD4+ T cells from three donors. Uninfected cells received control supernatant, infected cells received an inoculum of GFP- or DSredX-expressing HIV-1 NL4.3 and bystander cells are the GFP/DSredX-negative population from the infected cell samples. A. Intracellular staining for GLUT1 with antibody Ab40084 demonstrates that the level of GLUT1 expression within cells is not altered upon infection with HIV-1 NL4.3-GFP. B. Staining of surface expressed GLUT1 with GLUT1-RBD-GFP of primary CD4+ T cells upon infection with HIV-1 NL4.3-DSredX. C. Median fluorescence intensity of surface expressed GLUT1 on HIV-1 NL4.3-DSredX infected and uninfected primary CD4+ T cells. D. Upregulation of surface expressed GLUT1 upon activation of primary CD4+ T cells. Additional file 4:
**Monitoring glycolysis and oxidative phosphorylation in HIV-1 NL4.3 infected and uninfected CD4+ T cells upon provision of galactose.** A. The extracellular acidification rate (ECAR) and B. oxygen consumption rate (OCR) over time is given as a percentage of the baseline recorded at 41 minutes in the absence of glucose for primary CD4+ T cells from three donors, CEM-ss and Jurkat cells. Vertical lines indicate the injection of galactose and oligomycin, respectively. Data from primary CD4+ T cells represent single plates with 10 wells dedicated to each condition and data for cell lines CEM-ss and Jurkat are the average of two plates. Error bars represent the standard deviation and asterisks indicate p-values from an unpaired t-test: *0.01 < p < 0.05, **0.005 < p < 0.01, ***p < 0.005, data points without an asterisk have non-significant differences. Additional file 5:
**Cell proliferation of HIV-1 infected and uninfected primary CD4+ T cells in DMEM with galactose or glucose.** A. Cell proliferation of T cells as determined by CFSE dilution. Primary CD4+ T cells of donors 1 and 2 were stained with CFSE and cultured for 4 days in DMEM with 1 g/L of either galactose, glucose or a combination of both. At day 1 and day 4 cells were harvested and analysed by flow cytometry. B. Proliferation of primary CD4+ T cells from donors 1 and 2 that were infected with HIV-1 NL4.3 (HIV-1) or non-infectious Env-deleted HIV-1 NL4.3 (Δ Env) in DMEM containing 1 g/L galactose or glucose. Data for HIV-1 infected cells represent the population of cells that were gated to contain p24^Gag^-expressing cells only. Additional file 6:
**Cells infected with HIV-1 NL4.3 have a survival advantage when cultured in media containing galactose compared with glucose.** Primary CD4+ T cells from donors 4 and 5 were infected with HIV-1 NL4.3 in RPMI with IL-2. After 24 hours cells were washed and seeded into DMEM containing galactose, glucose or a combination of the two in the absence or presence of reverse transcriptase inhibitors (RTI). Cells were harvested at 24 hour intervals and analysed for intracellular HIV-1 p24^Gag^ expression by flow cytometry. Data are shown as the percentage of p24^Gag^ positive cells. Additional file 7:
**The survival advantage of infected primary CD4+ T cells cultured in media containing galactose compared with glucose is not an artifact of outgrowth of uninfected cells in cultures containing glucose.** The data shown are derived from those in Figure 4 and have been adjusted to represent actual cell numbers after counting with CountBright beads according to the manufacturer’s instructions. Additional file 8:
**Cells infected with VSV-G-pseudotyped HIV-1 YU2 have a survival advantage when cultured in media containing galactose compared with glucose.** A. Primary CD4+ T cells were infected with HIV-1 NL4.3 in RPMI with IL-2. After 24 hours cells were washed and seeded into DMEM containing galactose, glucose or a combination of the two in the absence or presence of reverse transcriptase inhibitors (RTI). Cells were harvested at 24 hour intervals and analysed for intracellular HIV-1 p24^Gag^ expression by flow cytometry. Data are shown as the percentage of p24^Gag^ positive cells. B. Percentage of p24^Gag^ positive Jurkat and CEM-ss cells cultured in DMEM containing galactose, glucose or a combination of the two in the absence or presence of reverse transcriptase inhibitors (RTI). C. The data shown are derived from those in A and have been adjusted to represent actual cell numbers after counting with CountBright beads according to the manufacturer’s instructions. Additional file 9:
**A greater proportion of cells infected with VSV-G-pseudotyped HIV-1 YU2 express activated caspase 3 when cultured in media containing glucose compared with galactose.** A. The ratio of activated caspase 3 positive to negative cells after 24 hours of culturing in media containing galactose or glucose for primary CD4+ T cells from 3 different donors and CEM-ss cells. B. The ratio of phosphorylated AKT1 (p-AKT1) positive to negative cells after 24 hours of culturing in media containing galactose or glucose for primary CD4+ T cells from 3 different donors and CEM-ss cells. Uninfected cells received an inoculum of control supernatant from cells that were transfected with the VSV-G expression plasmid only. Additional file 10:
**Primary CD4+ T cells cultured in glucose are more susceptible to apoptosis compared with galactose.** A. Scatter plots demonstrating the gating of HIV-1 NL4.3-GFP and annexin V positive populations of HIV-1 infected primary CD4+ T cells and CEM-ss cells. The data shown correspond to day 2 post infection, which represents 1 day of culturing in media containing 1 g/L galactose or glucose. B. The ratio of annexin V positive to negative cells after 24 hours of culturing in media containing galactose or glucose for primary CD4+ T cells from three different donors and CEM-ss cells. Additional file 11:
**Cytokine production in HIV-1 NL4.3 infected primary cells is unaffected by culturing in galactose or glucose subsequent to activation and infection.** Analysis of intracellular cytokine expression of HIV-1 infected cells after 24 hours of culturing in media containing galactose or glucose for primary CD4+ T cells from three different donors. A. Analysis of the intracellular expression of IL-2, IL-4 and IFNγ in HIV-1 NL4.3 infected cells that were gated on the p24^Gag^ positive population. The graphs indicate the percentages of IL-2, IL-4 and IFNγ single positive as well as dual positive populations as indicated on the right of the diagrams. B. The ratio of TNFα positive to negative cells after 24 hours of culturing in media containing galactose or glucose for primary CD4+ T cells from 3 different donors. The HIV-1 NL4.3 culture was subdivided into bystander cells and HIV-1 infected cells by gating for p24^Gag^. Note that TNFα expression in cells treated with HIV-1 NL4.3 ∆ Env were not determined (ND) for donor 1. Additional file 12:
**Early stages of HIV-1 replication are moderately affected in cultures containing galactose compared with glucose.** Cells cultured in DMEM containing 1 g/L galactose, glucose or a combination of the two were infected with HIV-1 NL4.3 and assessed for intracellular p24^Gag^ expression 24 hours later. The amount of p24^Gag^-positive cells was normalised to the values obtained for cultures containing glucose. Data represent eight independent infections with primary CD4+ T cells from five donors. Error bars represent the standard deviation and asterisks indicate p-values from ANOVA with Tukey post-test relating to the differences between cultures containing galactose only and cultures containing glucose or glucose and galactose: **0.005 < p < 0.01. Additional file 13:
**HIV-1 virion production is dependent on glycolysis.** A. Infectivity of culture supernatants from HIV-1 NL4.3 infected primary CD4+ T cells, Jurkat (Jur) and CEM-ss (CEM) cells cultured for 24 hours in media containing 4.5 g/L glucose or 1 g/L glucose and 3.5 g/L 2-deoxyglucose was determined by β-galactosidase activity of the indicator cell line TZM-bl. B. Western blots showing the presence of p24^Gag^ in the supernatant from cultures of HIV-1 NL4.3 infected primary CD4+ T cells, Jurkat and CEM-ss in media containing glucose (glc) or glucose and 2-deoxyglucose (2-DG). C. Quantified data from western blotting of p24^Gag^ shown in (B). All experiments were performed in triplicate and are shown as the average, normalised to the infectivity of cultures containing glucose and corrected for the number of live cells in the culture as determined with CountBright beads. Error bars represent the standard deviation.
