# Supplementary figures and images for: HIV-1 pathogenicity and virion production are dependent on the metabolic phenotype of activated CD4+ T cells
Source: Retrovirology. 2014 Nov 25;11:98. doi: 10.1186/s12977-014-0098-4 (PMC4252996; doi:10.1186/s12977-014-0098-4)

donor 1

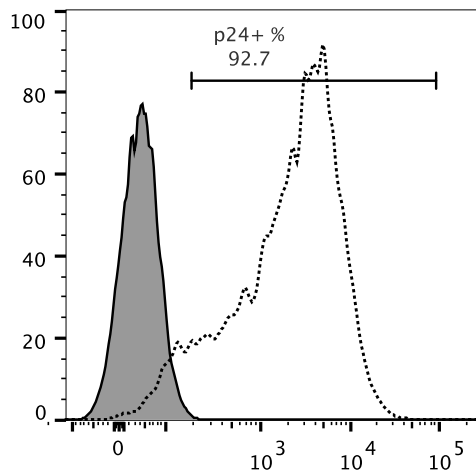

donor 2

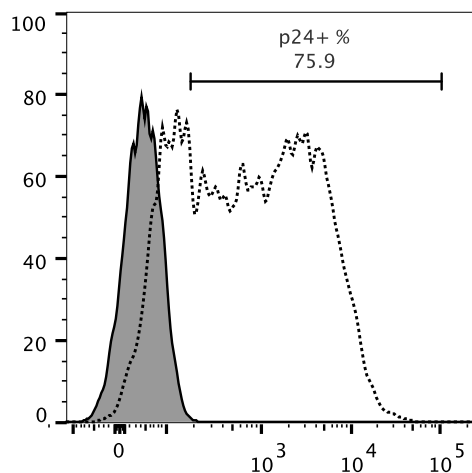

donor 3

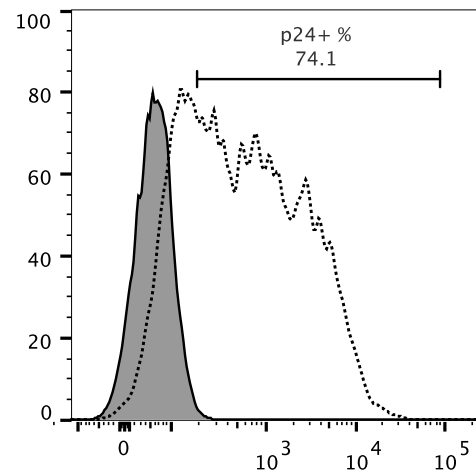

Jurkat

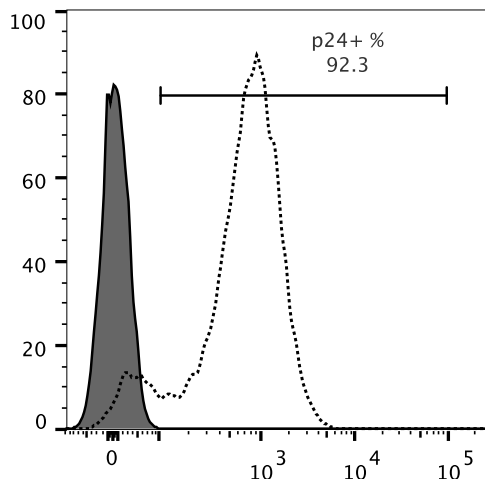

CEM-ss

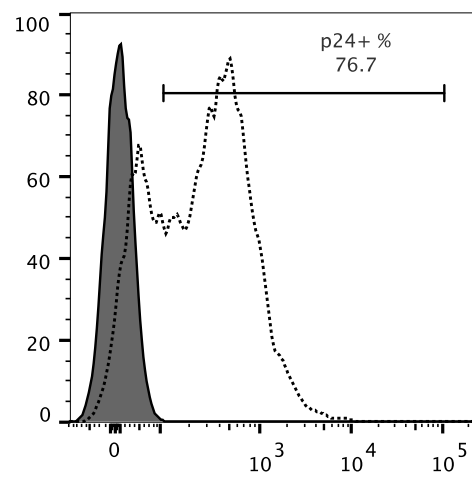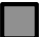 HIV-1 NL4.3 Δ Env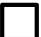 HIV-1 NL4.3 wt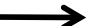 p24<sup>Gag</sup>

Supplement: Additional file 1: — Viral burden in infected primary CD4+ T cells and cells lines that were subjected to extracellular acidification and oxygen consumption experiments. Viral burden was assessed by intracellular staining of viral p24Gag antigen of HIV-1 NL4.3 wt infected cells compared with cells treated with NL4.3 Δ Env. A line on top of the plots indicates the gating boundaries for p24Gag-positive cells and the corresponding percentage of infected cells in the culture. [file 12977_2014_98_MOESM1_ESM.pdf]

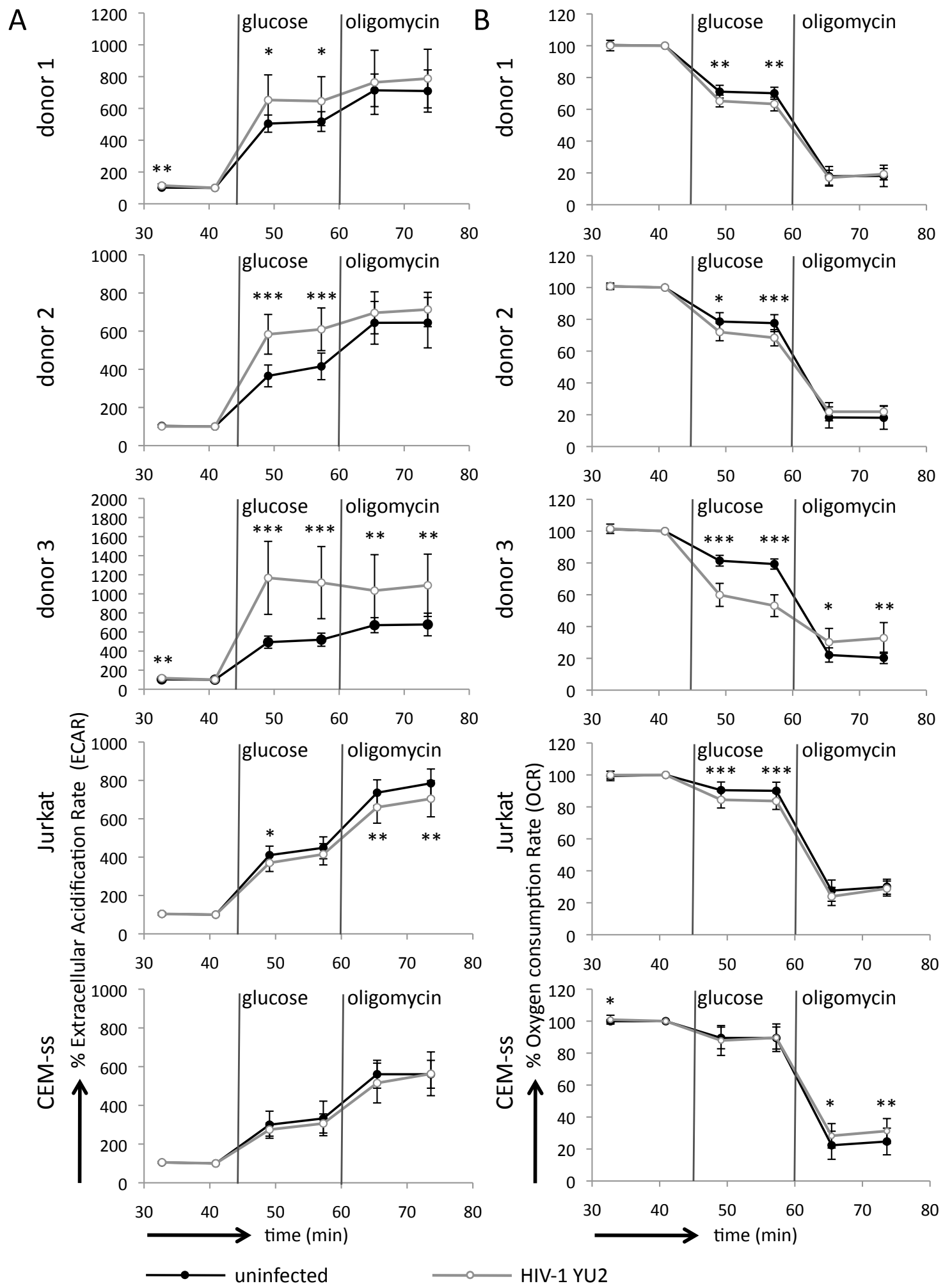

Supplement: Additional file 2: — Monitoring glycolysis and oxidative phosphorylation in VSV-G-pseudotyped HIV-1 YU2 infected and uninfected CD4+ T cells upon provision of glucose. A. The extracellular acidification rate (ECAR) and B. oxygen consumption rate (OCR) over time is given as a percentage of the baseline recorded at 41 minutes in the absence of glucose for primary CD4+ T cells from three donors, CEM-ss and Jurkat cells. Vertical lines indicate the injection of glucose and oligomycin, respectively. Data from primary CD4+ T cells represent single plates with 10 wells dedicated to each condition and data for cell lines CEM-ss and Jurkat are the average of three plates. In all cases, infected cell populations were at least 70% positive for HIV-1 p24Gag as determined by flow cytometry. Error bars represent the standard deviation and asterisks indicate p-values from an unpaired t-test: *0.01 < p < 0.05, **0.005 < p < 0.01, ***p < 0.005, data points without an asterisk have non-significant differences. [file 12977_2014_98_MOESM2_ESM.pdf]

A

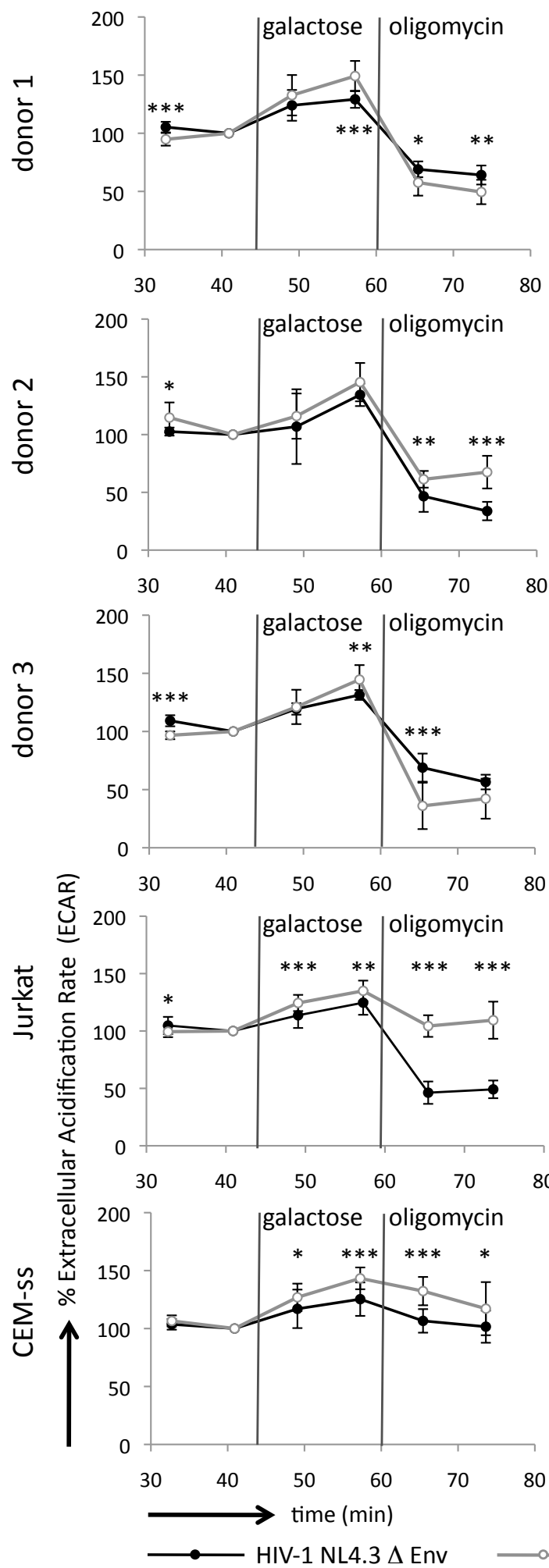

B

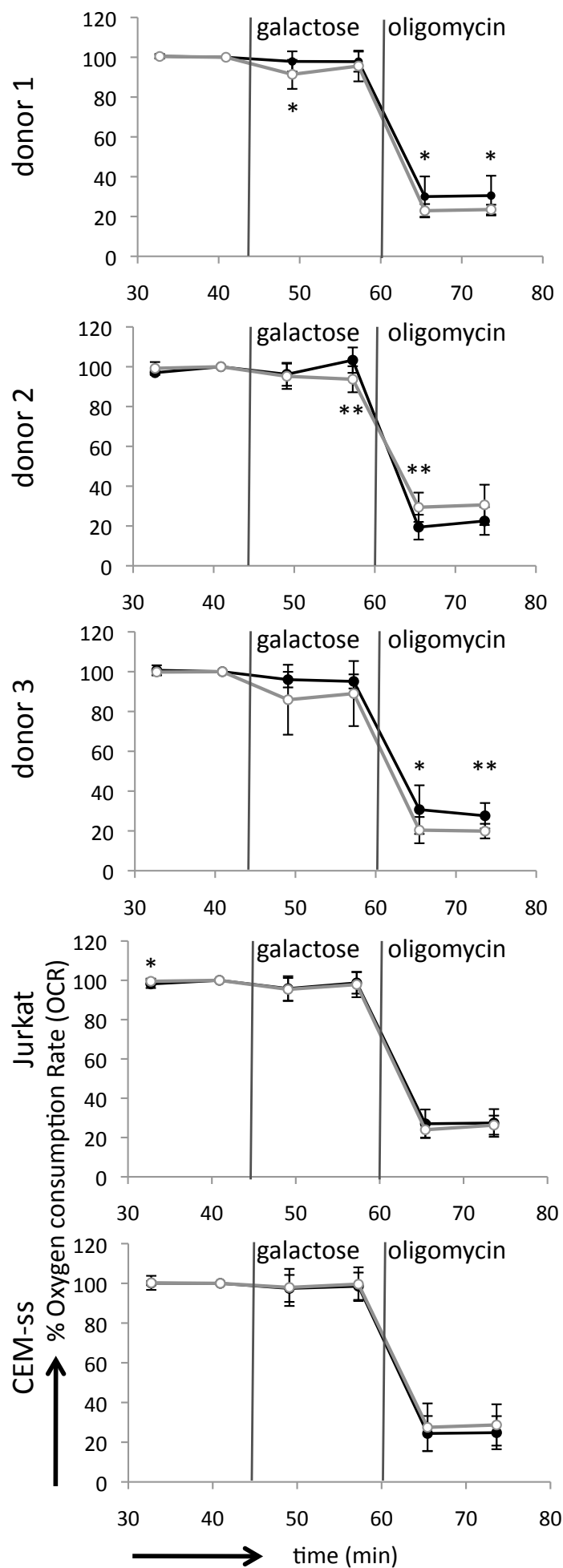

Supplement: Additional file 4: — Monitoring glycolysis and oxidative phosphorylation in HIV-1 NL4.3 infected and uninfected CD4+ T cells upon provision of galactose. A. The extracellular acidification rate (ECAR) and B. oxygen consumption rate (OCR) over time is given as a percentage of the baseline recorded at 41 minutes in the absence of glucose for primary CD4+ T cells from three donors, CEM-ss and Jurkat cells. Vertical lines indicate the injection of galactose and oligomycin, respectively. Data from primary CD4+ T cells represent single plates with 10 wells dedicated to each condition and data for cell lines CEM-ss and Jurkat are the average of two plates. Error bars represent the standard deviation and asterisks indicate p-values from an unpaired t-test: *0.01 < p < 0.05, **0.005 < p < 0.01, ***p < 0.005, data points without an asterisk have non-significant differences. [file 12977_2014_98_MOESM4_ESM.pdf]

**A**

donor 1

donor 2

day 1

day 4

→ CFSE

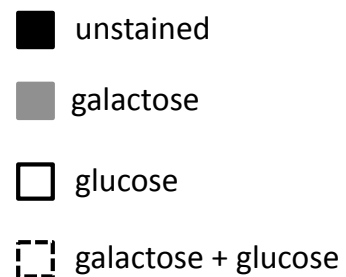**B**

day 4 post infection

→ CFSE

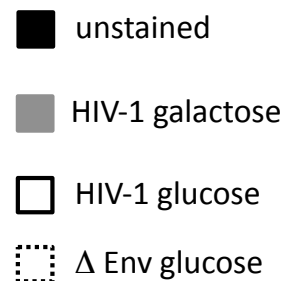

Supplement: Additional file 5: — Cell proliferation of HIV-1 infected and uninfected primary CD4+ T cells in DMEM with galactose or glucose. A. Cell proliferation of T cells as determined by CFSE dilution. Primary CD4+ T cells of donors 1 and 2 were stained with CFSE and cultured for 4 days in DMEM with 1 g/L of either galactose, glucose or a combination of both. At day 1 and day 4 cells were harvested and analysed by flow cytometry. B. Proliferation of primary CD4+ T cells from donors 1 and 2 that were infected with HIV-1 NL4.3 (HIV-1) or non-infectious Env-deleted HIV-1 NL4.3 (Δ Env) in DMEM containing 1 g/L galactose or glucose. Data for HIV-1 infected cells represent the population of cells that were gated to contain p24Gag-expressing cells only. [file 12977_2014_98_MOESM5_ESM.pdf]

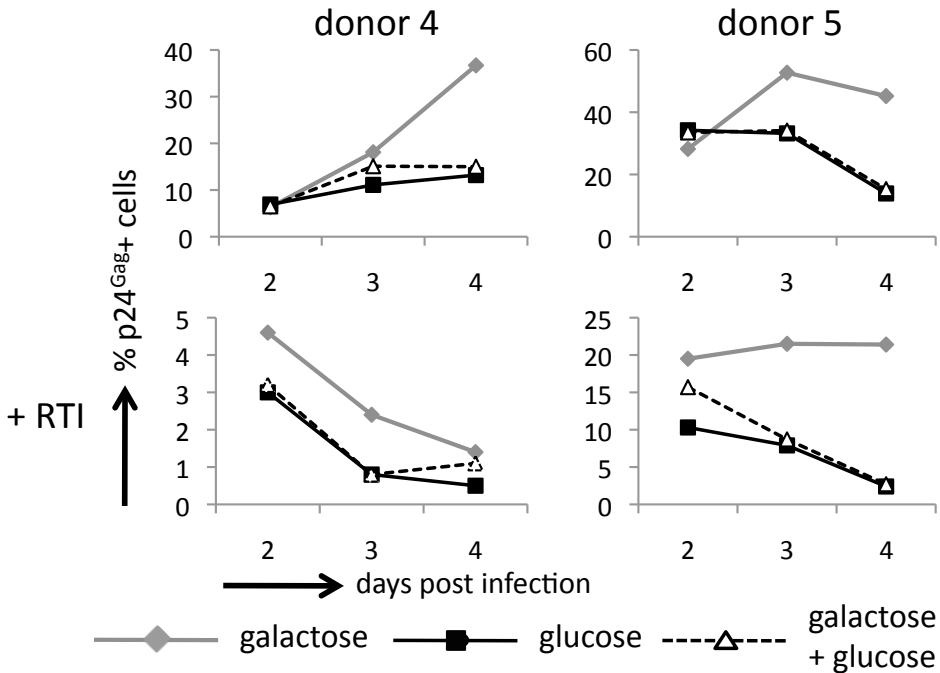

Supplement: Additional file 6: — Cells infected with HIV-1 NL4.3 have a survival advantage when cultured in media containing galactose compared with glucose. Primary CD4+ T cells from donors 4 and 5 were infected with HIV-1 NL4.3 in RPMI with IL-2. After 24 hours cells were washed and seeded into DMEM containing galactose, glucose or a combination of the two in the absence or presence of reverse transcriptase inhibitors (RTI). Cells were harvested at 24 hour intervals and analysed for intracellular HIV-1 p24Gag expression by flow cytometry. Data are shown as the percentage of p24Gag positive cells. [file 12977_2014_98_MOESM6_ESM.pdf]

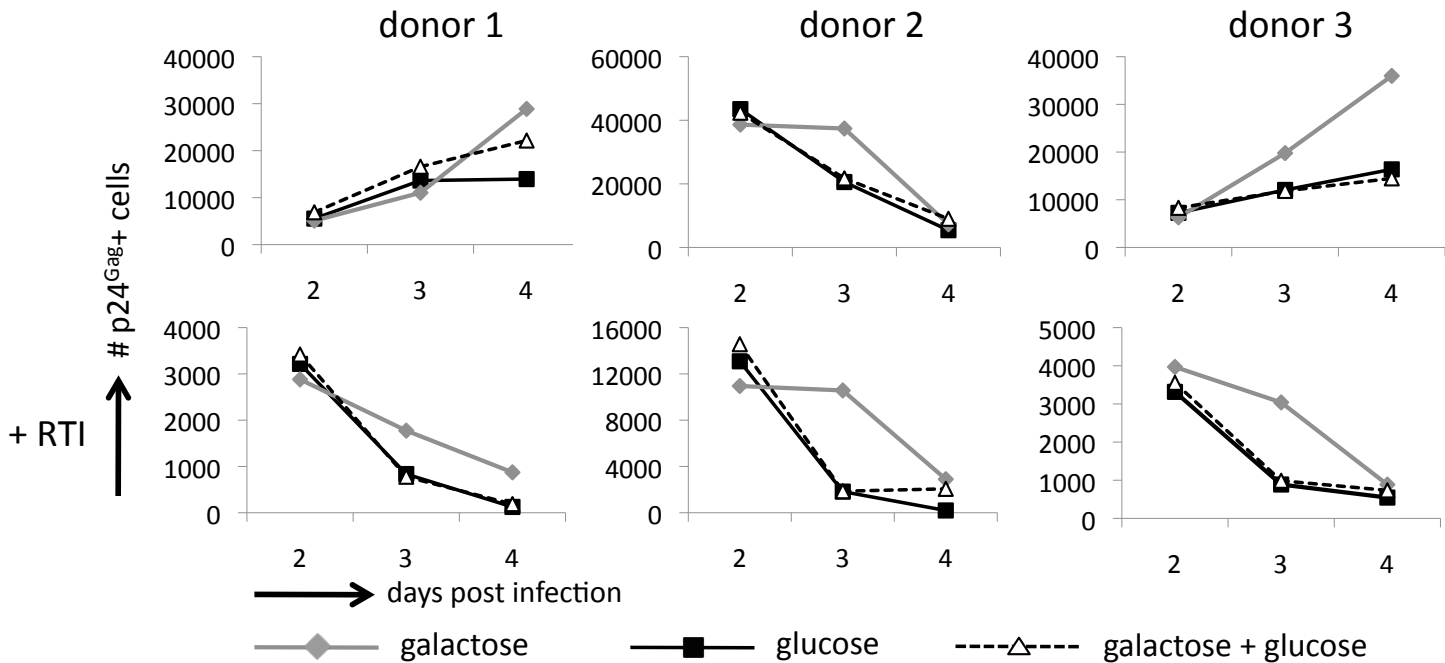

Supplement: Additional file 7: — The survival advantage of infected primary CD4+ T cells cultured in media containing galactose compared with glucose is not an artifact of outgrowth of uninfected cells in cultures containing glucose. The data shown are derived from those in Figure 4 and have been adjusted to represent actual cell numbers after counting with CountBright beads according to the manufacturer’s instructions. [file 12977_2014_98_MOESM7_ESM.pdf]

**A**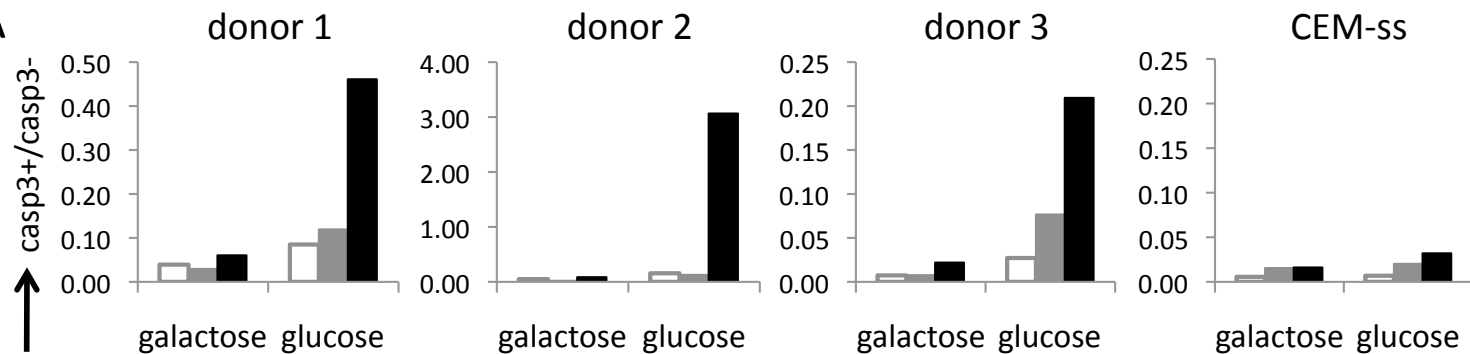**B**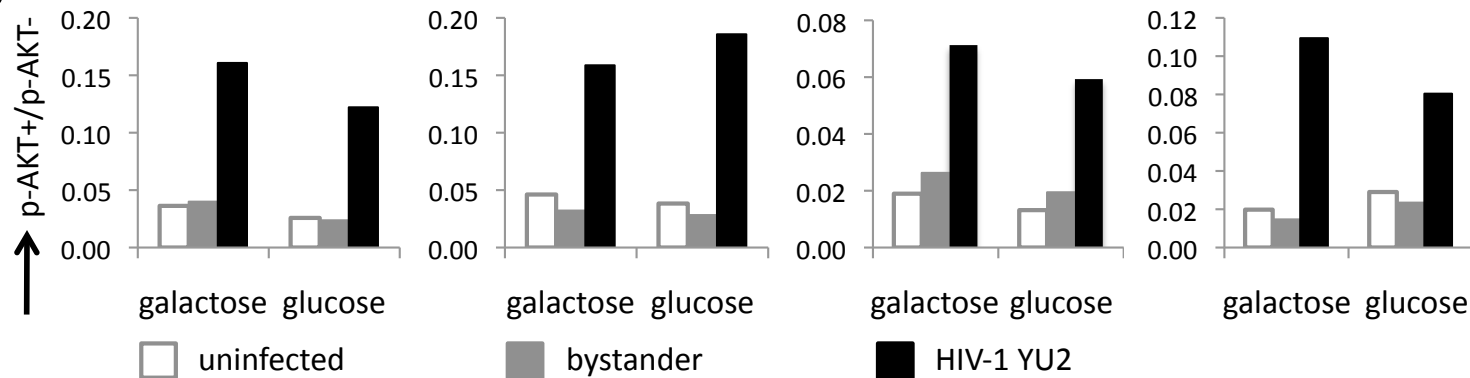

Supplement: Additional file 9: — A greater proportion of cells infected with VSV-G-pseudotyped HIV-1 YU2 express activated caspase 3 when cultured in media containing glucose compared with galactose. A. The ratio of activated caspase 3 positive to negative cells after 24 hours of culturing in media containing galactose or glucose for primary CD4+ T cells from 3 different donors and CEM-ss cells. B. The ratio of phosphorylated AKT1 (p-AKT1) positive to negative cells after 24 hours of culturing in media containing galactose or glucose for primary CD4+ T cells from 3 different donors and CEM-ss cells. Uninfected cells received an inoculum of control supernatant from cells that were transfected with the VSV-G expression plasmid only. [file 12977_2014_98_MOESM9_ESM.pdf]

**A**primary CD4<sup>+</sup> T cells

CEM-ss

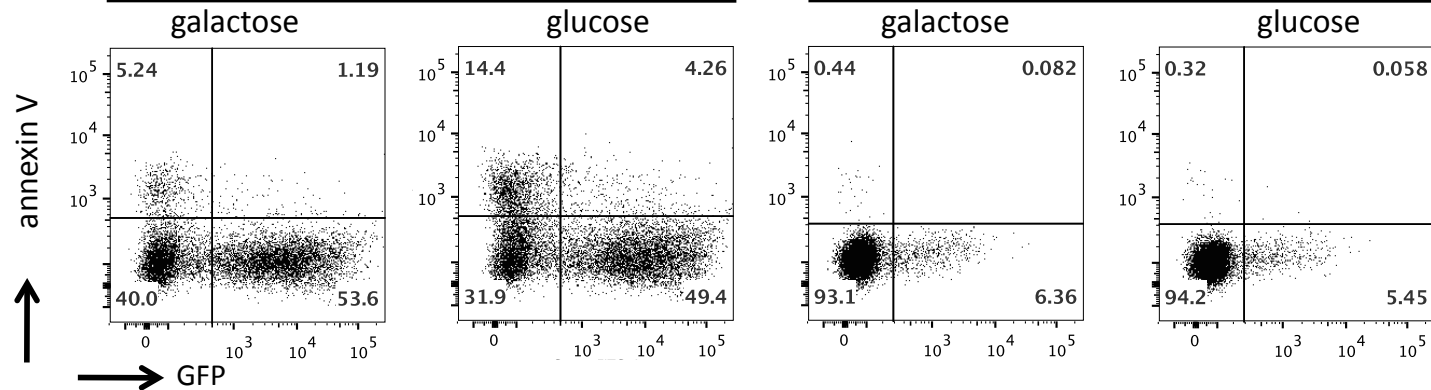**B**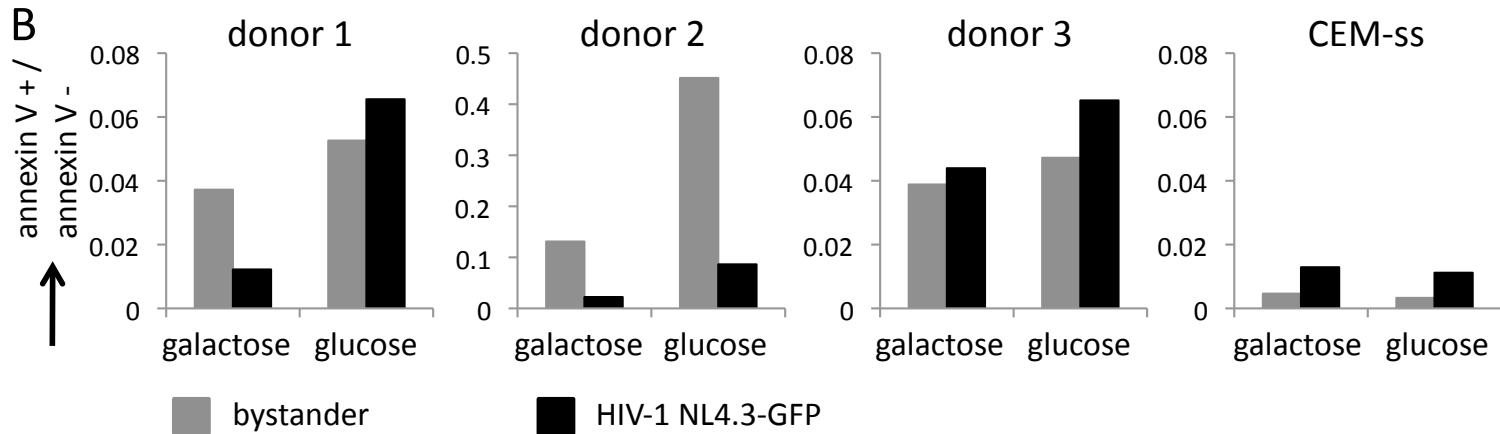

Supplement: Additional file 10: — Primary CD4+ T cells cultured in glucose are more susceptible to apoptosis compared with galactose. A. Scatter plots demonstrating the gating of HIV-1 NL4.3-GFP and annexin V positive populations of HIV-1 infected primary CD4+ T cells and CEM-ss cells. The data shown correspond to day 2 post infection, which represents 1 day of culturing in media containing 1 g/L galactose or glucose. B. The ratio of annexin V positive to negative cells after 24 hours of culturing in media containing galactose or glucose for primary CD4+ T cells from three different donors and CEM-ss cells. [file 12977_2014_98_MOESM10_ESM.pdf]

A

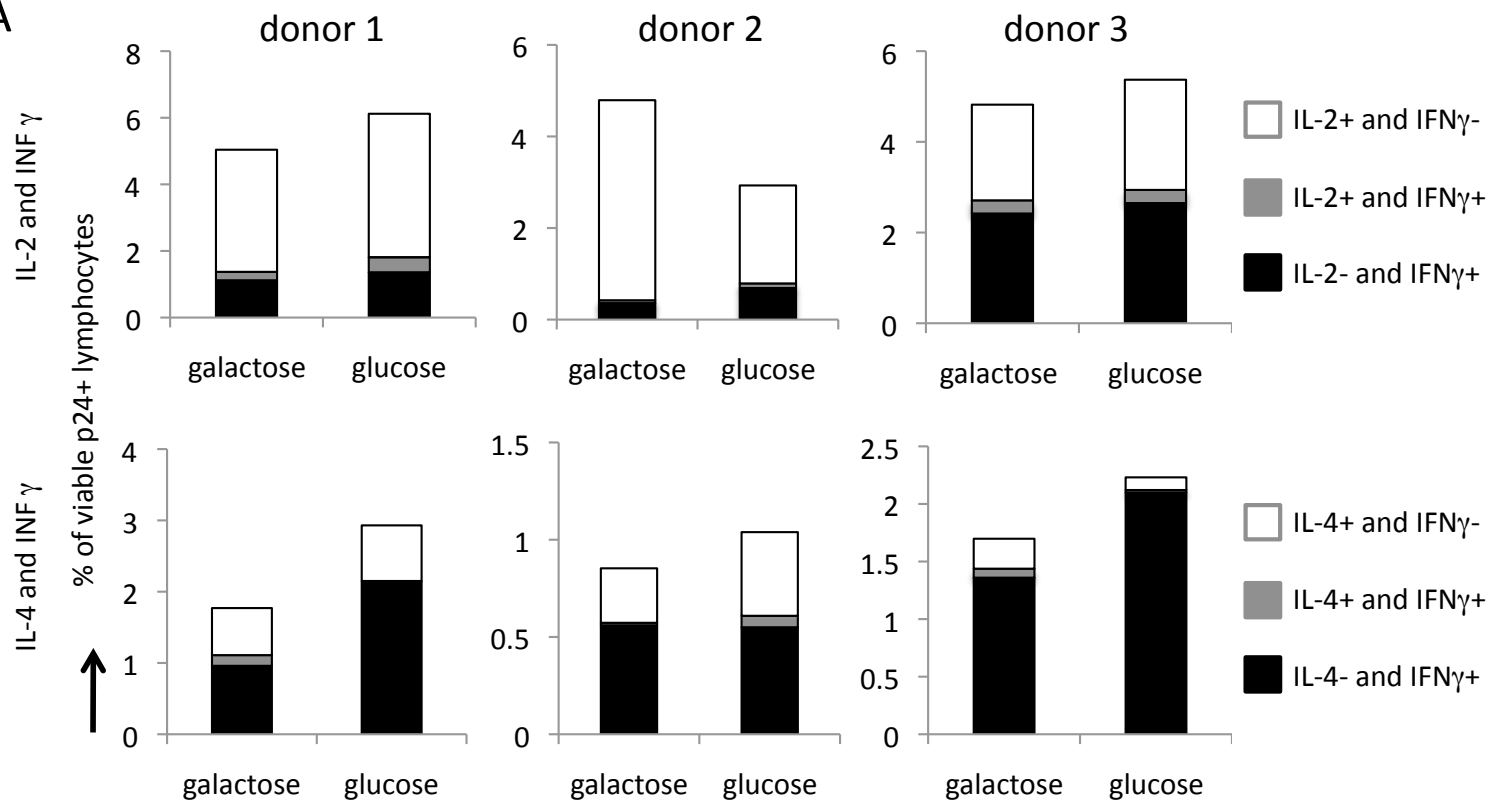

B

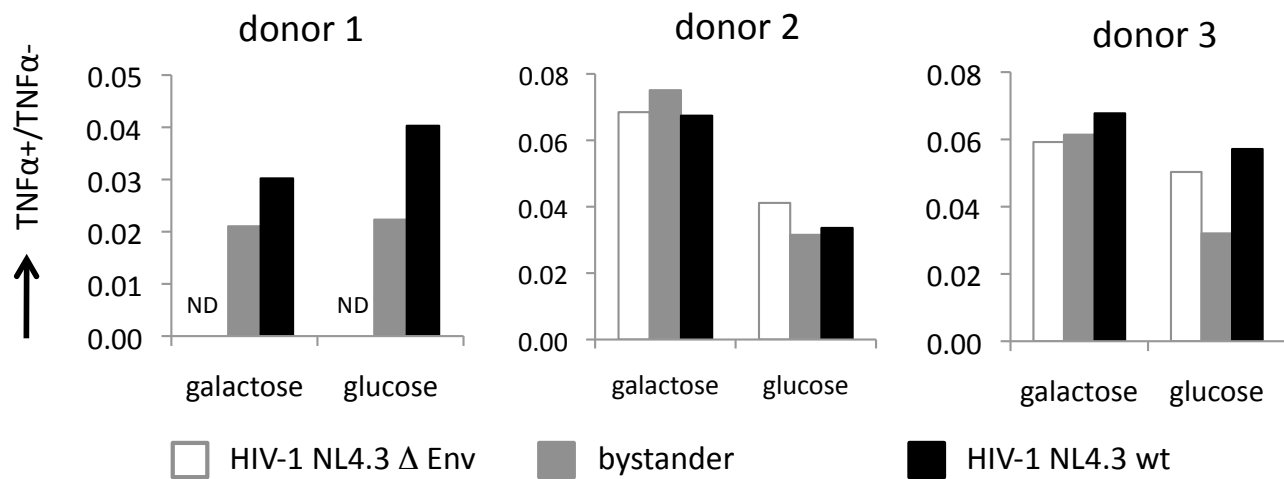

Supplement: Additional file 11: — Cytokine production in HIV-1 NL4.3 infected primary cells is unaffected by culturing in galactose or glucose subsequent to activation and infection. Analysis of intracellular cytokine expression of HIV-1 infected cells after 24 hours of culturing in media containing galactose or glucose for primary CD4+ T cells from three different donors. A. Analysis of the intracellular expression of IL-2, IL-4 and IFNγ in HIV-1 NL4.3 infected cells that were gated on the p24Gag positive population. The graphs indicate the percentages of IL-2, IL-4 and IFNγ single positive as well as dual positive populations as indicated on the right of the diagrams. B. The ratio of TNFα positive to negative cells after 24 hours of culturing in media containing galactose or glucose for primary CD4+ T cells from 3 different donors. The HIV-1 NL4.3 culture was subdivided into bystander cells and HIV-1 infected cells by gating for p24Gag. Note that TNFα expression in cells treated with HIV-1 NL4.3 ∆ Env were not determined (ND) for donor 1. [file 12977_2014_98_MOESM11_ESM.pdf]

relative p24<sup>Gag</sup>+ cells

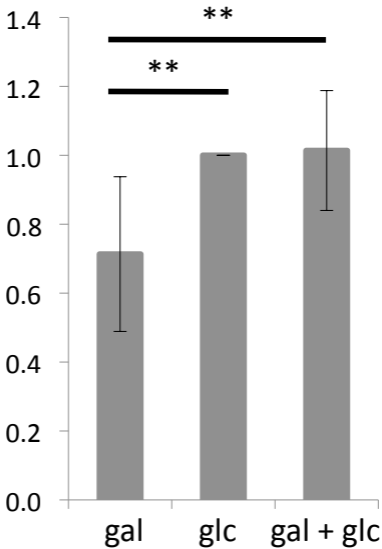

Supplement: Additional file 12: — Early stages of HIV-1 replication are moderately affected in cultures containing galactose compared with glucose. Cells cultured in DMEM containing 1 g/L galactose, glucose or a combination of the two were infected with HIV-1 NL4.3 and assessed for intracellular p24Gag expression 24 hours later. The amount of p24Gag-positive cells was normalised to the values obtained for cultures containing glucose. Data represent eight independent infections with primary CD4+ T cells from five donors. Error bars represent the standard deviation and asterisks indicate p-values from ANOVA with Tukey post-test relating to the differences between cultures containing galactose only and cultures containing glucose or glucose and galactose: **0.005 < p < 0.01. [file 12977_2014_98_MOESM12_ESM.pdf]

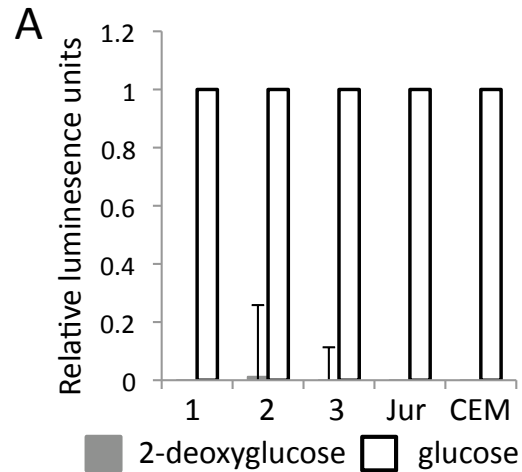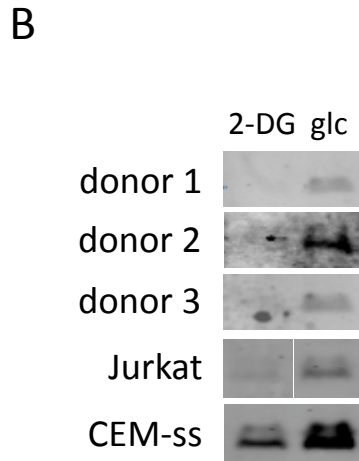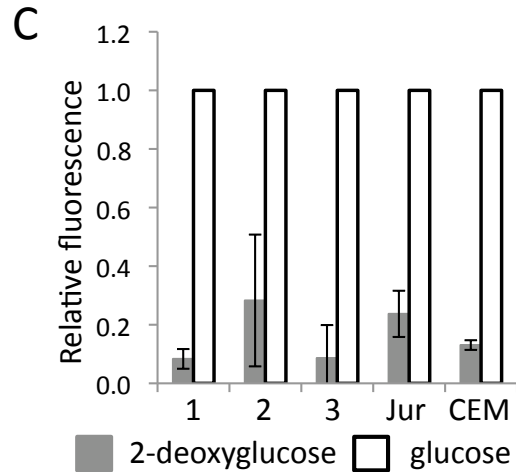

Supplement: Additional file 13: — HIV-1 virion production is dependent on glycolysis. A. Infectivity of culture supernatants from HIV-1 NL4.3 infected primary CD4+ T cells, Jurkat (Jur) and CEM-ss (CEM) cells cultured for 24 hours in media containing 4.5 g/L glucose or 1 g/L glucose and 3.5 g/L 2-deoxyglucose was determined by β-galactosidase activity of the indicator cell line TZM-bl. B. Western blots showing the presence of p24Gag in the supernatant from cultures of HIV-1 NL4.3 infected primary CD4+ T cells, Jurkat and CEM-ss in media containing glucose (glc) or glucose and 2-deoxyglucose (2-DG). C. Quantified data from western blotting of p24Gag shown in (B). All experiments were performed in triplicate and are shown as the average, normalised to the infectivity of cultures containing glucose and corrected for the number of live cells in the culture as determined with CountBright beads. Error bars represent the standard deviation. [file 12977_2014_98_MOESM13_ESM.pdf]
