# Supplementary material for: HIV-1 pathogenicity and virion production are dependent on the metabolic phenotype of activated CD4+ T cells
Source: Retrovirology. 2014 Nov 25;11:98. doi: 10.1186/s12977-014-0098-4 (PMC4252996; doi:10.1186/s12977-014-0098-4)

A

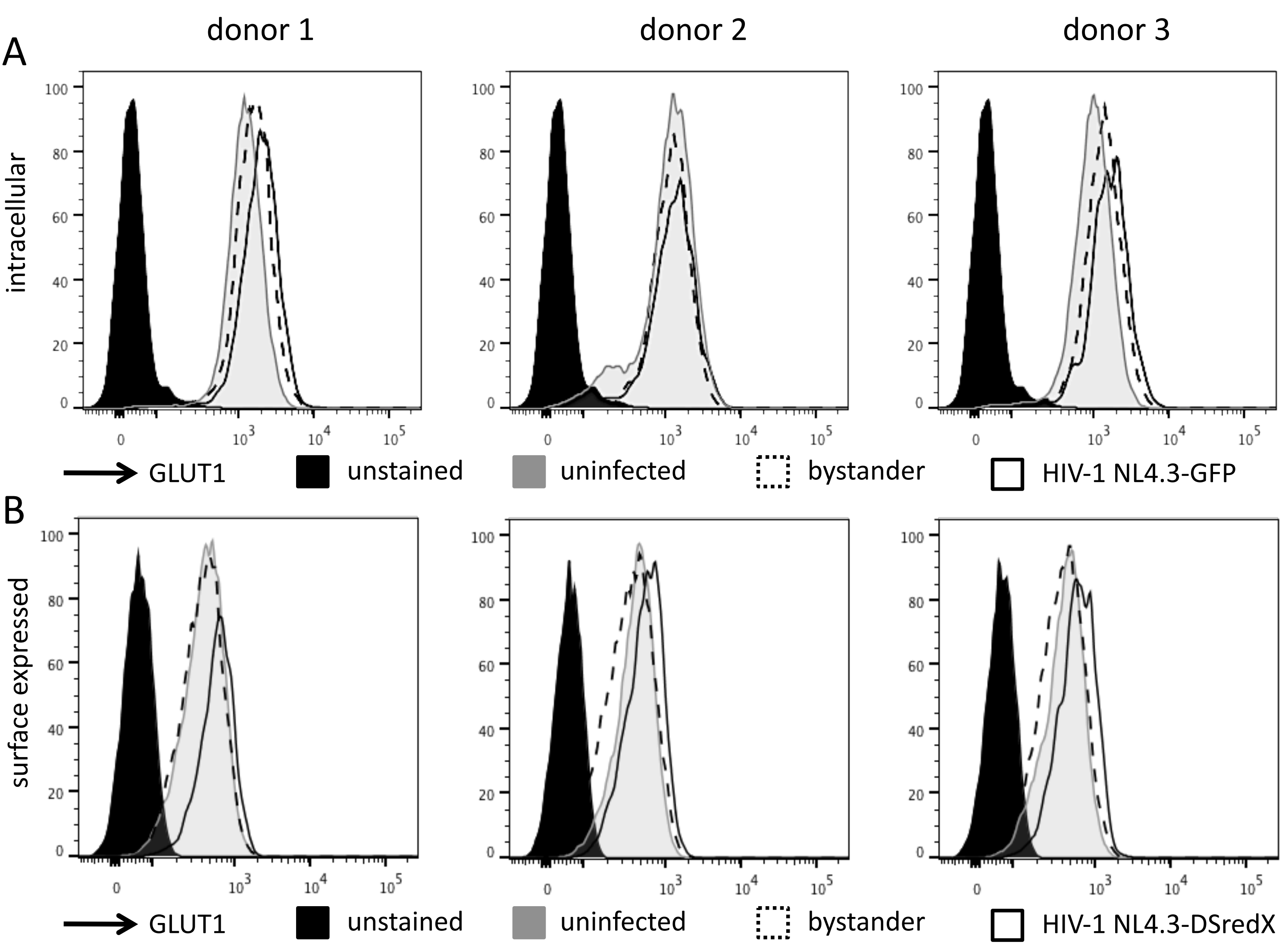

C

surface-expressed GLUT1	unstained	uninfected	bystander	infected
donor 1	69.5	527	501	746
donor 2	69.5	544	463	694
donor 3	78.2	533	492	732

median fluoresence intensity in relative fluoresence units

D

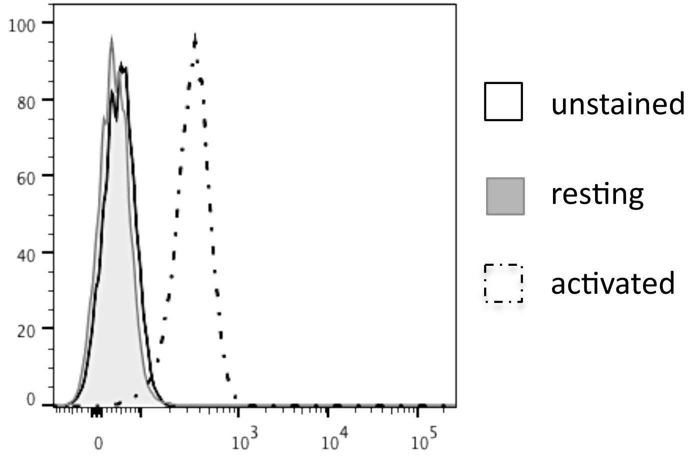

Supplement: Additional file 3: — Intracellular and surface GLUT1 expression in infection of primary CD4+ T cells infected with HIV-1 NL4.3-GFP/DSredX. Flow cytometry analysis of GLUT1 expression in primary CD4+ T cells from three donors. Uninfected cells received control supernatant, infected cells received an inoculum of GFP- or DSredX-expressing HIV-1 NL4.3 and bystander cells are the GFP/DSredX-negative population from the infected cell samples. A. Intracellular staining for GLUT1 with antibody Ab40084 demonstrates that the level of GLUT1 expression within cells is not altered upon infection with HIV-1 NL4.3-GFP. B. Staining of surface expressed GLUT1 with GLUT1-RBD-GFP of primary CD4+ T cells upon infection with HIV-1 NL4.3-DSredX. C. Median fluorescence intensity of surface expressed GLUT1 on HIV-1 NL4.3-DSredX infected and uninfected primary CD4+ T cells. D. Upregulation of surface expressed GLUT1 upon activation of primary CD4+ T cells. [file 12977_2014_98_MOESM3_ESM.pdf]
