# Supplementary material for: HIV-1 pathogenicity and virion production are dependent on the metabolic phenotype of activated CD4+ T cells
Source: Retrovirology. 2014 Nov 25;11:98. doi: 10.1186/s12977-014-0098-4 (PMC4252996; doi:10.1186/s12977-014-0098-4)
Supplement: Additional file 8: — Cells infected with VSV-G-pseudotyped HIV-1 YU2 have a survival advantage when cultured in media containing galactose compared with glucose. A. Primary CD4+ T cells were infected with HIV-1 NL4.3 in RPMI with IL-2. After 24 hours cells were washed and seeded into DMEM containing galactose, glucose or a combination of the two in the absence or presence of reverse transcriptase inhibitors (RTI). Cells were harvested at 24 hour intervals and analysed for intracellular HIV-1 p24Gag expression by flow cytometry. Data are shown as the percentage of p24Gag positive cells. B. Percentage of p24Gag positive Jurkat and CEM-ss cells cultured in DMEM containing galactose, glucose or a combination of the two in the absence or presence of reverse transcriptase inhibitors (RTI). C. The data shown are derived from those in A and have been adjusted to represent actual cell numbers after counting with CountBright beads according to the manufacturer’s instructions. [file 12977_2014_98_MOESM8_ESM.pdf]

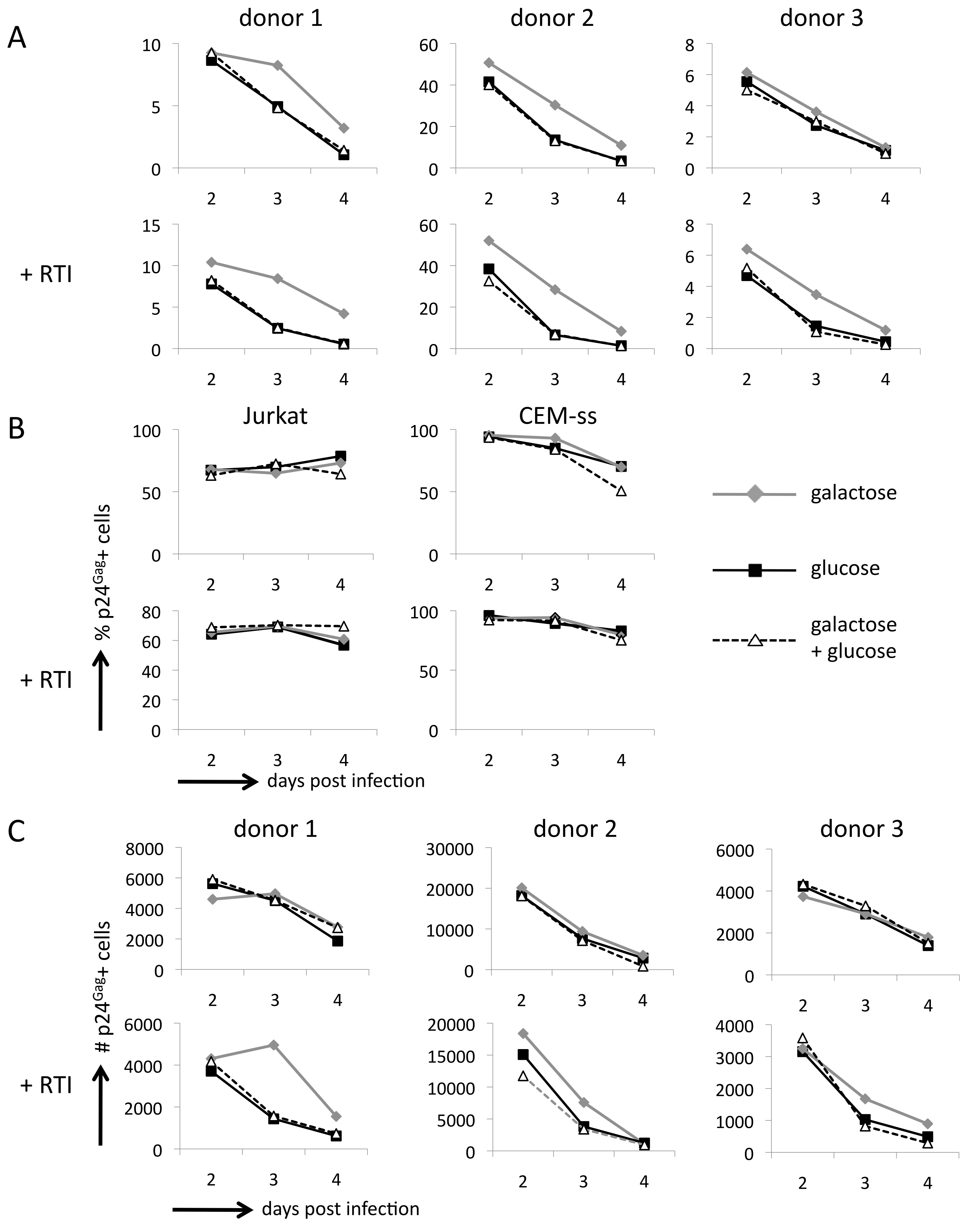

A
donor 1
donor 2
donor 3
+ RTI
B
Jurkat
CEM-ss
% p24Gag+ cells
days post infection
galactose
glucose
galactose + glucose
C
donor 1
donor 2
donor 3
# p24Gag+ cells
+ RTI
days post infection
